# A unified framework for packing deformable and non-deformable subcellular structures in crowded cryo-electron tomogram simulation

**DOI:** 10.1186/s12859-020-03660-w

**Published:** 2020-09-09

**Authors:** Sinuo Liu, Xiaojuan Ban, Xiangrui Zeng, Fengnian Zhao, Yuan Gao, Wenjie Wu, Hongpan Zhang, Feiyang Chen, Thomas Hall, Xin Gao, Min Xu

**Affiliations:** 1 Beijing Advanced Innovation Center for Materials Genome Engineering, School of Computer and Communication Engineering, University of Science and Technology Beijing, Beijing, China; 2 Computational Biology Department, Carnegie Mellon University, Pittsburgh, PA United States; 3 WuYuzhang Honors College, Sichuan University, Sichuan, China; 4 Beijing, China; 5 School of Information Science and Technology, Beijing Forestry University, Beijing, China; 6 College of Life Science, Sichuan University, Sichuan, China; 7 Thuwal, Saudi Arabia, King Abdullah University of Science and Technology (KAUST), Computational Bioscience Research Center (CBRC), Computer, Electrical and Mathematical Sciences and Engineering (CEMSE) Division, Thuwal, Saudi Arabia; 8 School of Mechanical, Electrical and Information Engineering, Shandong University, Shandong, China

**Keywords:** Cryo-electron tomography, Unified packing, Coarse-graining, Molecular dynamics

## Abstract

**Background:**

Cryo-electron tomography is an important and powerful technique to explore the structure, abundance, and location of ultrastructure in a near-native state. It contains detailed information of all macromolecular complexes in a sample cell. However, due to the compact and crowded status, the missing edge effect, and low signal to noise ratio (SNR), it is extremely challenging to recover such information with existing image processing methods. Cryo-electron tomogram simulation is an effective solution to test and optimize the performance of the above image processing methods. The simulated images could be regarded as the labeled data which covers a wide range of macromolecular complexes and ultrastructure. To approximate the crowded cellular environment, it is very important to pack these heterogeneous structures as tightly as possible. Besides, simulating non-deformable and deformable components under a unified framework also need to be achieved.

**Result:**

In this paper, we proposed a unified framework for simulating crowded cryo-electron tomogram images including non-deformable macromolecular complexes and deformable ultrastructures. A macromolecule was approximated using multiple balls with fixed relative positions to reduce the vacuum volume. A ultrastructure, such as membrane and filament, was approximated using multiple balls with flexible relative positions so that this structure could deform under force field. In the experiment, 400 macromolecules of 20 representative types were packed into simulated cytoplasm by our framework, and numerical verification proved that our method has a smaller volume and higher compression ratio than the baseline single-ball model. We also packed filaments, membranes and macromolecules together, to obtain a simulated cryo-electron tomogram image with deformable structures. The simulated results are closer to the real Cryo-ET, making the analysis more difficult. The DOG particle picking method and the image segmentation method are tested on our simulation data, and the experimental results show that these methods still have much room for improvement.

**Conclusion:**

The proposed multi-ball model can achieve more crowded packaging results and contains richer elements with different properties to obtain more realistic cryo-electron tomogram simulation. This enables users to simulate cryo-electron tomogram images with non-deformable macromolecular complexes and deformable ultrastructures under a unified framework. To illustrate the advantages of our framework in improving the compression ratio, we calculated the volume of simulated macromolecular under our multi-ball method and traditional single-ball method. We also performed the packing experiment of filaments and membranes to demonstrate the simulation ability of deformable structures. Our method can be used to do a benchmark by generating large labeled cryo-ET dataset and evaluating existing image processing methods. Since the content of the simulated cryo-ET is more complex and crowded compared with previous ones, it will pose a greater challenge to existing image processing methods.

## Background

Cryo-electron tomography (Cryo-ET), which was first proposed in 1970s, is now a popular and powerful imaging technique in the fields of life and medical sciences [1–3]. A series of two-dimensional images recorded by electron microscopes are collected to generate 3D reconstruction of macromolecules and then used to analyze the architecture of these structures. In traditional sample preparation process, due to the incompatibility with vacuum, water in sample cells tends to boil out and leads to unacceptable explosions. This seriously effected the efficiency of the sample preparation process and the accuracy of result data. In order to extract the cellular structure more accurately with higher resolution, cryo-electron tomography has emerged, which prepares samples at low temperatures and is able to record the cellular structure in a natural state [4].

In the fields of protein visualization and structural biology, various machine learning methods are applied to the analyse the structure of macromolecules and ultrastructures [5–7]. These methods can resolve the structure of macromolecules to a large extent, but the accuracy still need to be improved. On one hand, due to the lack of training data, only unsupervised methods could be introduced. These models often fail to obtain credible results and can only make fuzzy estimates. On the other hand, the parameter adjustment process is very time consuming, and it is extremely difficult to verify whether these specific parameters can obtain the best results [8, 9]. Therefore, generating simulated cryo-ET is very important. To make the simulation result more realistic, Molecular Dynamics(MD) is introduced. The label of simulated cryo-ET data is known which is very helpful in training machine learning algorithms and improving the performance. It is also of great help to adjust parameters and find the optimal ones.

There are many studies on the simulation of the interaction and movement of subcelluar structures. Some studies focus on the simplified modeling of these structures [10–13] and the generation of topology file [14]. Others tend to explore the interaction between a pair of structures [15, 16]. In this field, the study of macromolecular crowding is of great importance[17]. In 2015, Pei et al. [18] proposed to simulate cryo-ET of macromolecular crowding. This method modeled macromolecules with single ball, and squeezed them into a limited space. This method can simulate crowded cell cytoplasm at varying crowding levels by a score function. Random noise is used to achieve varying SNR levels. However, representing a macromolecule with only one sphere can not obtain the tightest packing results.

In general, traditional packing methods tend to model a single structure with a single cubic, sphere, cylinder, or other three-dimensional box. A specific macromolecule is placed in the smallest box that can hold it, which successfully simplified the complex into an alternative with regular boundaries. Although these models are easier to operate for the experimenter, only rough results can be obtained due to the huge waste of space. For example, when modeling a stick-like substance, the radius of the minimum boundary ball is extremely large due to its large height to thickness ratio. This means a large area of vacuum is present inside the sphere. This kind of model cannot represent the stick-like substance and its surrounding environment accurately during the simulation process. In contrast, a cuboid may be more suitable for simulating this type of structure. Besides, the scale of the minimum boundary is fixed, so it cannot exhibit the deformation and stretching of structures. The packing result is not tight enough because it is limited by the space waste issue. The result of the simulation is not rich enough because it lacks the dynamic features. These inherent problems with existing methods lead to the inaccurate representation of the cytoplasmic environment, and it hardly provides adequate assistance to subsequent image process methods. To this end, a new scheme to simulate deformable ultrastructure, such as filaments and membranes, and non-deformable macromolecules, like proteins, in a unified framework is of great significance. It is also very important to achieve the result of crowded packing, which is based on the movement and interaction of the above structure according to molecular dynamics. This method is not only a supplement and development of traditional biological structure modeling, but also a great challenge in computer graphics, expecially physics-based simulation [19–21].

In the field of protein visualization, many basic methods have been proposed to simplify a macromolecule which composed of thousands of atoms based on their structural information. These methods are collectively referred to as coarse-grained methods, which include the efficient and feasible clustering methods. Classical clustering methods include hierarchical methods [22], partition-based methods [23], density-based methods [24], grid-based methods [25], and model-based methods [26]. Specifically, partition-based methods are simple and efficient for large data sets. This type of methods has a low space complexity which means it requires less time than other methods. The k-means method [27], as a representative partition-based method, allows the user to freely set the number of clusters and divides the data according to the similarity between discrete points. When used with macromolecules, the clustered atoms are close in distance, and the positions of the atoms can be used effectively as the basis for dividing the atoms into clusters.

In this paper, we proposed a unified simulation framework for packing deformable and non-deformable structures together under a rather crowded status. The non-deformable macromolecules are coarse-grained by the k-means clustering method. A limited number of fixed-position spheres with different radii are used to represent a single macromolecule. Deformable filament and membrane are modeled by multiple balls with flexible relative positions. Generally speaking, filaments and membranes are relatively larger than ordinary maceomolecules, and they are more flexible in interactions and movement modes. The number of balls in a specific structure is depending on its atom number. Next, the deformability of various structures is achieved by designing different typologies. For example, balls in a macromolecule will be fully connected to each other and the length of bonds is hard to change. This will make the relative position of the balls relatively fixed, and the macromolecule will reflect the rigid body characteristics. The filaments and membranes may have relatively few bonds which by using a not fully connected topology. The angle between two bonds is changeable to achieve deformable properties. Then, certain number of macromolecules, filaments, and membranes are placed in a given space, and be moved towards the center by applying a external force pointing to the center. After a certain period of time, the system reaches a sufficient crowding level. The movement process under bonded forces, non-bonded forces, and external forces follows molecular dynamics and conforms to physical laws.

## Method

The simulation of Cryo-electron tomogram with rigid macromolecules and deformable ultrastructure contains three parts: the modeling of macromolecules and ultrastructure, molecular dynamics simulation, and generating simulated cryo-electron tomograms. In this section, we will demonstrate the above three parts respectively.

### Modeling of macromolecules and ultrastructure

To simulate the inter-cellular environment realistically, the modeling of intercellular ultrastructures and macromolecule complexes is required. Traditional simulation methods tend to model a structure with single-element model, and do not take into account the deformable filament or membrane. This greatly reduces the richness of simulation results, and cannot represent the compact Cryo-electron tomography properly.

Therefore, we proposed to represent a macromolecule or ultrastructure with multiple-ball model which is able to describe deformable and non-deformable body in a unified framework, and could also save the space. This process is also called “coarse graining”. A high-resolution structure consisting of thousands of atoms is represented at a low resolution by limited number of balls. A topology file is also required to describe how a structure is organized, including the atomic mass and bond configurations. In this paper, the macromolecule, which is non-deformable, is approximated using multiple balls with fixed relative positions; while the deformable ultrastructure, like membranes and filaments,are approximated using multiple balls with flexible relative positions. This feature is achieved by setting different force fields for balls, and setting the chemical bond angle to have different elasticity.

#### Coarse graining of macromolecule

There are thousands of atoms in one macromolecule, which makes it extremely difficult to accurately represent a macromolecule with a limited number of balls. In this paper, we start with the coordinates of atoms and use the k-means method, a classical clustering algorithm in machine learning, to realize the coarse granulation of macromolecules. The algorithm of k-means method could be found in supplementary document, Section S2.

To improve the richness and accuracy of simulation, 20 macromolecules with different scales and shapes were selected from the Protein Data Bank (PDB) [28]. These PDB files contain abundant information about the complex such as the source, structure, sequence, etc. In a PDB file, the most important value that we are focused on is the spatial position of each atom, called the coordinates.

Clustering methods are unsupervised learning methods which is used to devide the data into several groups. It group objects with similar properties into the same cluster. It has a wide range of applications and can be applied to almost all objects. In k-means clustering method, k denotes that we want *k* clusters, and mean represents that we calculate the center of the cluster using the mean value of the attribute values in each cluster. In a word, the cluster is described by the centroid of each cluster. The more similar the objects in the same cluster are, the better the clustering accuracy is. Cluster analysis attempts to find the similarity between different objects, which is usually represented by their Euclidean distance, cosine distance, or Hamming distance. Obviously, for a macromolecule the Euclidean distance is a simple and effective way to group atoms which are close to each other into one cluster. In this paper, the clustering process was implement based on the k-means program in scikit-learn package [29].

In this paper, the k-means method is used to divide the atoms in a macromolecule into *k* clusters. First, the coordinates of all *n* atoms is extracted from the PDB file: *atoms*={*coord*_1_,*coord*_2_,...,*coord*_*n*_}, where *coord*_*i*_={(*x*_*i*_,*y*_*i*_,*z*_*i*_)∣*x*_*i*_,*y*_*i*_,*z*_*i*_∈*R*}. Then *k* atoms is randomly selected as the initial cluster center: *centers*_*tmp*_={*center*_1_,*center*_2_,...*center*_*k*_}, where *center*_*i*_={(*x*_*i*_,*y*_*i*_,*z*_*i*_)∣(*x*_*i*_,*y*_*i*_,*z*_*i*_)∈*atoms*}. Secondly, for each atom *i* in the macromolecule, the distance to all *k* centers *dist*_*i*_={*dist*_*i*→1_,*dist*_*i*→2_,...,*dist*_*i*→*k*_} is calculated by Euclidean Distance formula:

1 $$\begin{array}{@{}rcl@{}} dist_{i\rightarrow j}\! & \,=\, & \!\mid \mid coor_{i} - center_{j} \mid \mid \\ & \,=\, & \sqrt[2]{\left(x_{i}\,-\,x_{j}\right)^{2} \,+\, \left(y_{i}\,-\,y_{j}\right)^{2} \,+\, \left(z_{i}\,-\,z_{j}\right)^{2} } \end{array} $$

Next, each atom is assigned to the nearest cluster by finding the minimum value in its distant list ${dist_{i_{min}} = min\left (dist_{i}\right)}$. Then, the centroid of each cluster is recalculated, and *k* new cluster centers is obtained by the arithmetic mean:

2 $$ center_{i} = \frac{1}{N} \cdot {\sum\limits_{k=1}^{N} coord_{ik}}  $$

where *i* denotes the number of center, *coord*_*ik*_=(*x*_*k*_,*y*_*k*_,*z*_*k*_) is the coordinate of the *k*_*th*_ atom in cluster *i*. Then repeat the second and third steps to redistribute the atoms into different clusters until the cluster center no longer changes. Finally, in coarse graining process, the center of the *k* small balls is represented by the coordinates of the cluster center, and the radius is the maximum distance ${dist_{i_{max}} = max(dist_{i})}$ from the cluster center to the atom in this cluster. The radius of the small balls in a macromolecule is in the range of 20-65 $\overset {\circ }{A}$, most of which are concentrated between 35-55 $\overset {\circ }{A}$. The overall size of the macromolecule is around 60-250 $\overset {\circ }{A}$, most of which is concentrated between 120-160 $\overset {\circ }{A}$.

Due to the variety of macromolecule sizes,the number of atoms contained in each macromolecule is also different. Therefore, a uniformed cluster number *k* can not meet the need of reasonable coarse granulation. In this paper, the original number of cluster clusters *k*_*tmp*_ is set base on the scale of the macromolecule by the following formula:

3 $$ k_{tmp} = \lceil \frac{N_{atom}}{5000} \rceil   $$

where *N*_*atom*_ refers to the atom number of a macromolecule. This means that every five thousand atoms will be represented by one ball. When there are less than 5,000 remaining atoms, one ball will still be added to ensure that all particles are represented. It is important to make sure that each macromolecule is divided into at least three clusters to get three cluster centers. This means that each macromolecule can form at least two linearly independent vectors to determine the rotation angle of the macromolecule in space during subsequent molecular dynamics simulation step. Eq. 4 is used to limit the range of *k*:

4 $$ k = \left\{ \begin{array}{llll} 3 & & {k_{tmp} < 3}\\ k_{tmp} & & {k_{tmp} \geq 3} \end{array} \right.   $$

**Fig. 1:**
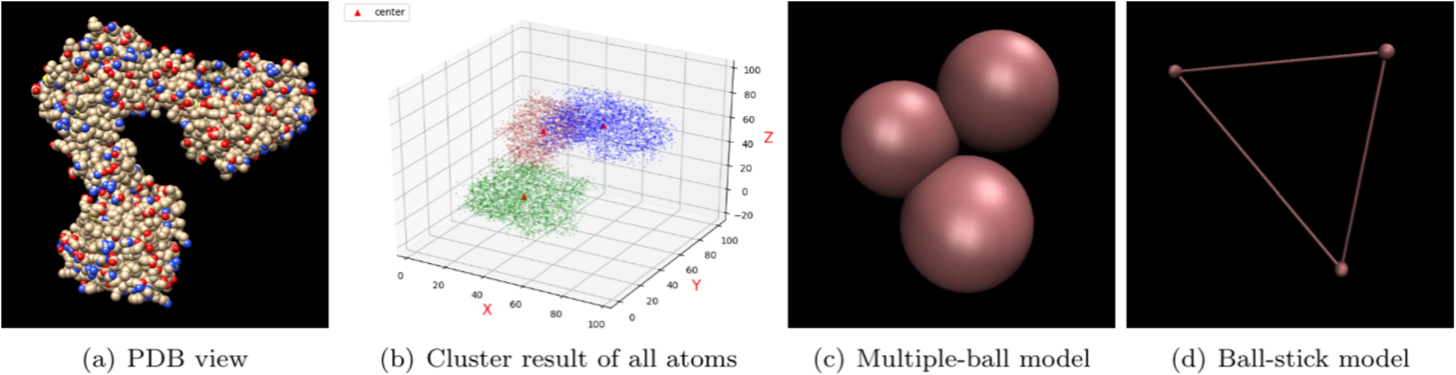
The coarse graining process of a macromolecule (macromolecule 1F1B). Subfigure (**a**) shows all atoms in macromolecule 1F1B. As shown in (**b**), all of the atoms in 1F1B are separated into 3 clusters using k-means clustering method. Three cluster centers are represented by red triangles, and the atoms in different clusters are marked by different colors. Our methods represent each macromolecule with several balls, and the 3D visualization result of our multiple-ball model on 1F1B is shown in subfigure (**c**). All the balls in a macromolecule are connected with each other. This fully connected topology guarantees its non-deformable nature. The topological structure of 1F1B is shown in (**d**) with a ball-stick model


The process of coarse graining is shown in Fig. 1, its algorithm is shown in Algorithm 1.

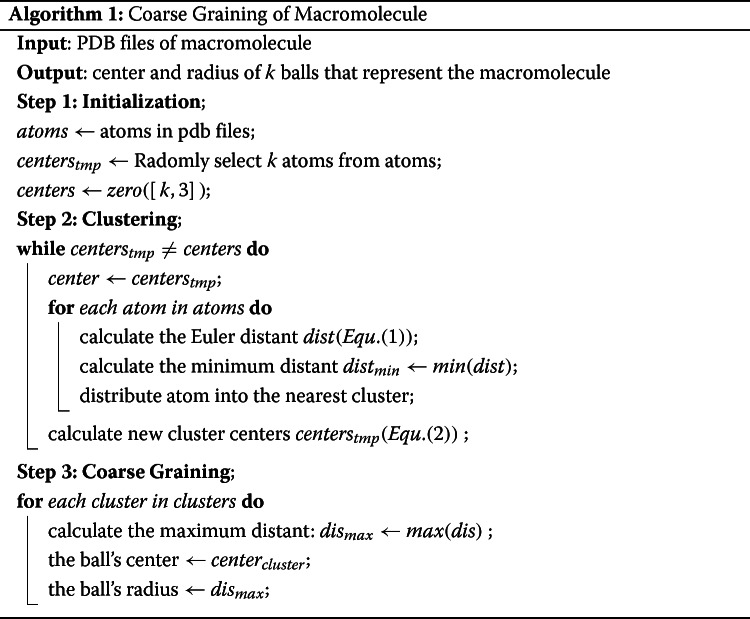


#### Model of filaments and membrane

The single ball/cylindrical model is difficult to properly simulate a large-scale filament or membrane. In contrast, the multi-ball model can not only decrease the vacuum volume, but also flexibly represent the shape change of the above structure by changing the relative position of the balls. In order to reduce the complexity of the model, the filament and membrane was initialized by the simplest model with a rule layout.

**Fig. 2:**
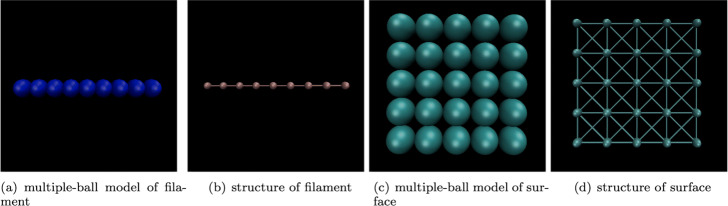
The initial multiple-ball model and topological structure of deformable structure (filament and surface/membrane). The initial model of a deformable structure is its simplest status. As shown in (**a**) and (**c**), all the balls are collinear in filament, while are coplanar in membrane. In our model, all balls in the same structure have the same radius. In order to represent deformable property, the topography of filaments and membranes are no longer similar to the fully connected macromolecule structure. As shown in (**b**), the balls in a filament is connected to its left and right neighbors in turn, and each ball is constrained by at most two chemical bonds. As shown in (**d**), balls in a membrane are connected to its surrounding four neighbors and form a mesh-like structure. For the above topology, as long as the bond length of the chemical bond not change, the structure will not stretch. Deformation such as bending can occur, when the bond angle changes elastically

For the filament, its initial state was set to a straight line, arranged by *m* balls. The model is shown in Fig. 2a. For the membrane, its initial state was set to a rectangular mesh, arranged by *m*∗*n* balls. The model is shown in Fig. 2c.

#### Topological structure

The deformability of a specific macromolecules or ultrastructure is determined by its topology structure. For macromolecules, we set the structure to be in-deformable. *k* spheres that make up the macromolecule are connected in pairs (see Fig. 1d), and the angles of the chemical bonds are fixed. For filament and membrane, we set the structure to be deformable. The angles of the chemical bonds is able to change elastically.

In a filament, inside balls are sequentially connected to the nearest neighbors of the left and right by the serial number. As shown in Fig. 2b, only balls at both ends are bound by one chemical bond, while the other balls are bound by two chemical bonds.

In a membrane, balls are in a mesh structure, and each ball is connected to the neighbors in the four directions of the upper, lower, left, and right directions. It is also connected to the neighbors of the four diagonal directions. The structure of a membrane is shown in Fig. 2d, the balls at the apex are bound by three chemical bonds, the balls at the boundary are bound by five chemical bonds, and the inner balls are bound by eight chemical bonds.

### Molecular dynamics simulation

The setting of the biomolecules force field is the most important part of dynamics simulation process. In this paper, force field could be divided into three types: bonded force, non-bonded force and external force. In each time step, all the particles move under the above forces. As for a specific structure, if the relative position of inner particles change significantly, this macromolecular complex will deform. For each ball *i* in the force field, its acceleration *a*_*i*_ at time *t* is described by the following formula:

5 $$\begin{array}{@{}rcl@{}} a_{i}(t) & = & \frac{1}{m} \cdot {F_{i}(t)} \\ & = & \frac{1}{m}\left({F_{i}}^{b}(t) + {F_{i}}^{nb}(t) + F_{i}^{ext}(t)\right) \end{array} $$

where *m* denodes the mass of each ball, *t* is the time, *F*_*i*_(*t*) is the resultant force, *F*_*i*_^*b*^(*t*), *F*_*i*_^*nb*^(*t*) and ${F_{i}^{ext}(t)}$ represent the bonded force, non-bonded force and external force respectively. Since each ball represents 5,000 atoms, the mass *m* in our experiment was set to 5000. Then the velocity and position of each ball is describe by the following foluma:

6 $$ v_{i}(t) = a_{i}(t)\cdot \delta t \\ \text{and} \\ x_{i}(t) = v_{i}(t)\cdot \delta t  $$

where *δt* is the time step, and it was set to 1 ns in this paper.

In biomelecules system, the force is calculated by negative gradient of the scalar potential function. The force from particle *j* at position *x*_*j*_ to particle *i* at position *x*_*i*_ is *F*(*x*_*ij*_)=−∇*U*(*x*_*ij*_), where *x*_*ij*_=*x*_*i*_−*x*_*j*_ is the position vector. Then Eq. 5 can be written as:

7 $$ a_{i}(t) = \frac{1}{m} \cdot \left(-\nabla \left(U_{i}^{b}(t) + U_{i}^{nb}(t) \right) + F_{i}^{ext}(t)\right)  $$

where ${ U_{i}^{b}(t)}$ is the bonded potential function, ${U_{i}^{nb}(t)}$ is the nonbonded potential function. The calculation of the above functions is implemented by NAnoscale Molecular Dynamics(NAMD) [30].

#### External force field

Our packing method considers external force which is a force field toward the center of simulation scene. The external forces is applied directly to the particles without the use of potential function. This force field lead all particles to move toward the center of the scene and squeeze together, which plays a major role in the packing operation.

In order to speed up the simulation process while maintaining the stability of the calculation in the crowded area, a force field action radius is set. Outside this radius, balls move toward the center under a uniform constant max force action. In order to avoid instability, the force field was set to be evenly decremented, and is reduced to zero in the center of the scene. The segmented external force field for ball *i* is as follows:

8 $$ F_{i}^{ext} = \left\{ \begin{array}{llll} 10\cdot \mid \mid x_{i}\mid \mid & & {\mid \mid x_{i}\mid \mid < 300}\\ 3000 & & {300 \leq \mid \mid x_{i}\mid \mid} \end{array} \right.   $$

where ∣∣*x*_*i*_∣∣ is the distance from ball *i* to the center of simulation scene.

#### Bond interactions

**Fig. 3:**
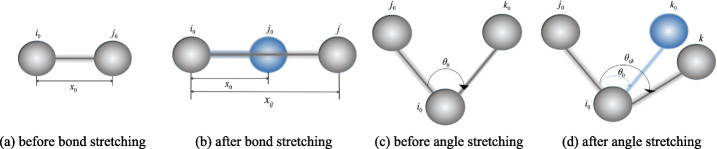
The bonded interactions. Subfigure (**a**) and (**b**) is the bond between ball *i* and ball *j* before and after streching. This interaction is constrict by Eq. 10 and plays a major role in the length change of chemical bonds. Subfigure (**c**) and (**d**) shows the angle streching of two bond between ball *i*, *j* and *k*. This interaction is constrained by Eq. 11 and is used to describe the bending of a structure

Bond interactions involve two type of constraint: bond stretching and angle stretching. As seen in Fig. 3, they are used to describe the 2-body and 3-body interaction of covalency bonded atoms respectively. The bond potential could be written as:

9 $$ U_{i}^{b} = \sum\limits_{j} U_{ij}^{bond} + \sum\limits_{j,k} U_{ijk}^{angle}  $$

For a pair of bonded particles *i* and *j*, their bond harmonic vibrational motion is described by spring bond potential:

10 $$ U_{ij}^{bond} = k_{b} \left(\mid \mid x_{ij}\mid \mid - \mid \mid x_{0}\mid \mid \right)^{2}   $$

where *k*_*b*_ is the spring constant and it was set to 2000, ∣∣*x*_*ij*_∣∣=∣∣*x*_*i*_−*x*_*j*_∣∣ is the distance between particle *i* and *j*, ||*x*_0_|| is the equilibrium distance of this particle pair. In this paper, ∣∣*x*_0_∣∣ of two specific bonded balls is set as the initial distance between two ball centers.

For three bonded particle *i*, *j* and *k*, their angular vibrational motion is described by angular bond potential:

11 $$ U_{ijk}^{angle} = k_{\theta} \left(||\theta_{ijk}|| - ||\theta_{0}||\right)^{2}   $$

Where *k*_*θ*_ is the angle constant, *θ*_*ijk*_ is the angle formated by vectors *x*_*ji*_=*x*_*j*_−*x*_*i*_ and *x*_*ki*_=*x*_*k*_−*x*_*i*_, *θ*_0_ is the equilibrium angle. In this paper, *θ*_0_ of is set as the initial angle of three ball centers.

Given the atomic coordinates and topology, the bonded constraint is the mainly influence factor of the deformation properties of macromolecules and ultrastructures. For a macromolecule, its topological structure is relatively stable because there are chemical bonds between any two balls in it. This means macromolecules do not deform during simulation as long as the length of the chemical bond is not changed. Therefore, *k*_*b*_ in the bond potential function is set to 2000, which is a very large value, to effectively ensure the rigidity of the macromolecule. In this paper, filament and membrane are set to deformable and non-stretchable structures. Therefore, *k*_*b*_ for filament and membrane is also set to 2000 to ensure the non-stretch feature, and *k*_*θ*_ is set to zero to enable the deformability of the angle.

It is obvious that the deformability of a structure is determined by its topological structure and bond constraint. The stability of the topological structure can determine the feasibility of the stretching and bending feature; the bond constraint is used to adjust the difficulty of the deformation.

#### Nonbond interactions

For particles pair *i* and *j* without chemical bond, van der Waals force play the main role of the interaction between them. There is a strong core repulsion between particle *i* and particle *j* if they are too close together, while there is a weak dipole attraction between them if they are a little far apart. The potential function of van der Waals force is described by Lennard–Jones potential:

12 $$ U_{ij}^{LJ} = \varepsilon \left[ \left(\frac{R_{min}}{||x_{ij}||} \right)^{12} - 2 \left(\frac{R_{min}}{||x_{ij}||} \right)^{6} \right]  $$

where *ε* is the depth of potential wall with the value ${-U_{ij}^{van}(R_{min}) }$, *R*_*min*_ is the distance at which the potential reach its minimum. This potential approaches 0 rapidly as ||*x*_*ij*_|| increases, so a switchdist and a cutoff point are chosen to save the computational resource. In this paper, the switchdist and cutoff are set to *S*=600*nm* and *C*=610*nm* respectively. This is a piecewise force field function. When the distance between the two particles is smaller than *S*, ${U_{ij}^{nb} = U_{ij}^{LJ} }$. Then, it starts to drop evenly from point *S* and become zero at point *C*.

In biomelecules system, the electrostatic force is also an important part of interactions. In this paper, since all the balls represent (0,5000] atoms, it is hard to say which sign of charge does it have. Besides, the goal of this paper is to pack a number of macromolecules into a limited space. Under the given external force, there is no need to calculate the inter-molecular forces accurately. Van der Waals force is enough to describe non-bond interactions and prevent particles form overlapping. In order to save computing resources, the charge of all balls is set to zero, and the electrostatic force is ignored.

### Generating simulated cryo-electron tomograms

To generate a simulated cryo-electron tomogram, we need to obtain the displacement vector and rotation angle of all the structures first. In this paper, each structure is approximated by multiple balls and represented by multiple vectors. For a structure *S* with *k* balls *B*={*b*_1_,*b*_2_,...,*b*_*k*_}, its position *coord*_*S*_=(*x*_*S*_,*y*_*S*_,*z*_*S*_) could be calculated by the arithmetic mean of *k* balls:

13 $$ \left\{ \begin{array}{llll} x_{S} & = & (x_{b1} + x_{b2} + \cdots + x_{bk})/k\\ y_{S} & = & (y_{b1} + y_{b2} + \cdots + y_{bk})/k \\ z_{S} & = & (z_{b1} + z_{b2} + \cdots + z_{bk})/k \end{array} \right.   $$

where *b*_*i*_=(*x*_*bi*_,*y*_*bi*_,*z*_*bi*_) is the coordination of the *i*_*th*_ ball center. The displacement vector is the vector from the initial position to the final position, that is ${\vec {V}_{d} = coord_{S}^{init} - coord_{S}^{final} }$.

**Fig. 4:**
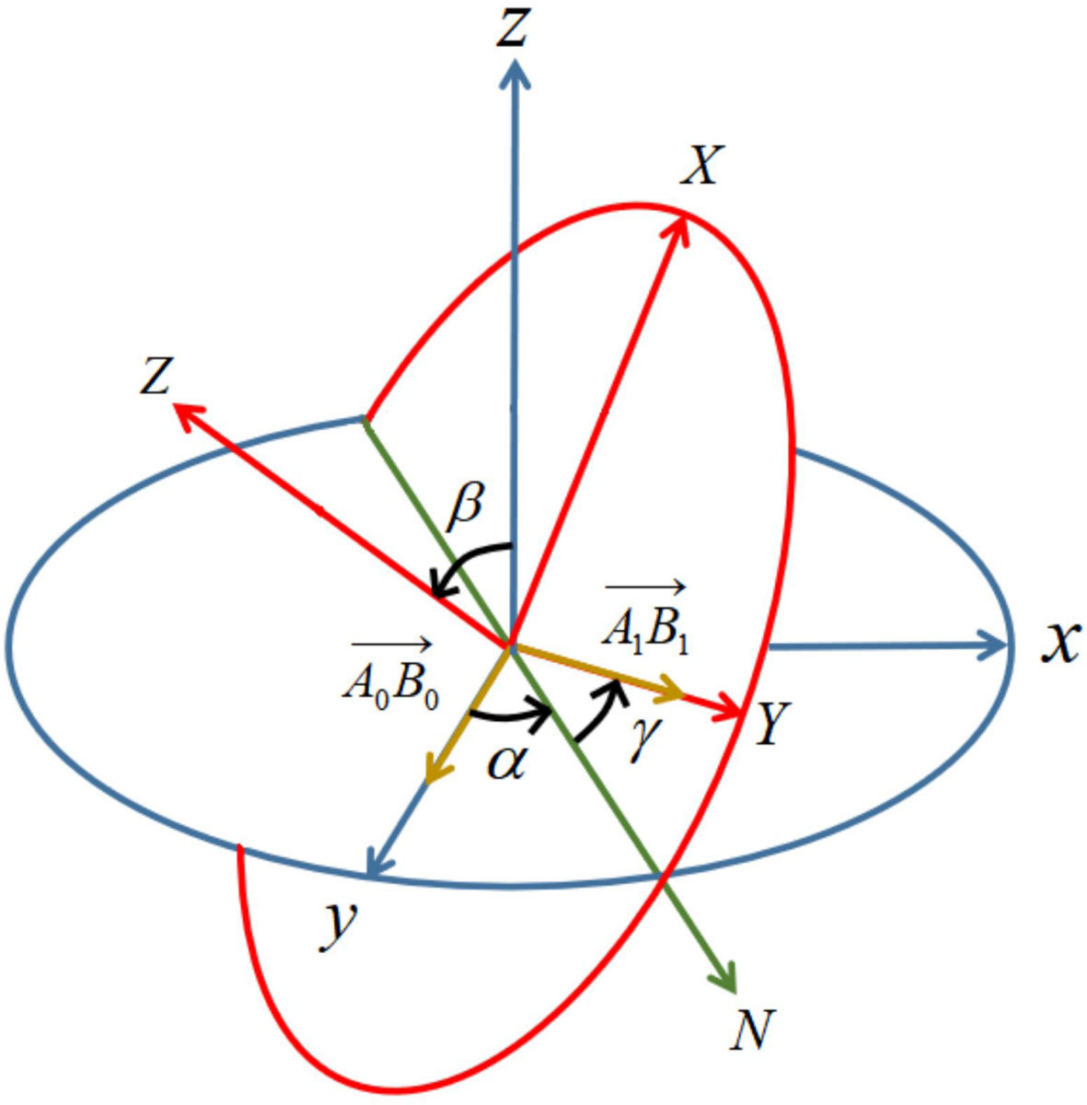
The Euler angle. In the three-dimensional space, vector *A*_0_*B*_0_ is rotated to the vector *A*_1_*B*_1_. In order to obtain the Euler angle, the coordinate system is rotated in accordance with the *ZYZ* format. The blue coordinate system is the initial state *XYZ*_*blue*_. The *XYZ*_*blue*_ axis is rotated by an angle *α* around the *Z* axis to obtain an intermediate coordinate system *XYZ*_*green*_. Then *XYZ*_*green*_ is rotated around its own *Y*-axis by an angle *β* to obtain the intermediate coordinate system *XYZ*_*red*_, at which time the blue *Z*-axis moves to the red *Z*-axis, and the *X*-axis and *Y*-axis are on the red plane. Rotate *XYZ*_*red*_ around its *Z* axis by angle *γ* to obtain the final coordinate system *XYZ*_*yellow*_, which is the red coordinate system in this figure. The rotation angle of the vector *A*_0_*B*_0_ to *A*_1_*B*_1_ is (*α*, *β*, *γ*)

The rotation of a point can be uniquely determined by a Euler angle which is represented by a triple array [31]. As shown in Fig. 4, the rotation accordance in this paper is *ZYZ* sequence, and the final rotation can be represented by a rotation matrix:

14 $$ R = R_{z}(\alpha)R_{y}(\beta)R_{z}(\gamma)  $$

where *α*, *β* and *γ* are the rotation angle in three sub-process.

For a rigid body in three-dimensional space, its rotation states can be uniquely determined by two linearly independent vectors inside it, and the rotation angle is obtained from the rotation matrixes of two vectors. Therefore, at least three points within each macromolecule need to be labeled, which is why each macromolecule need to consist of at least three clusters in the coarse graining process.

**Fig. 5:**
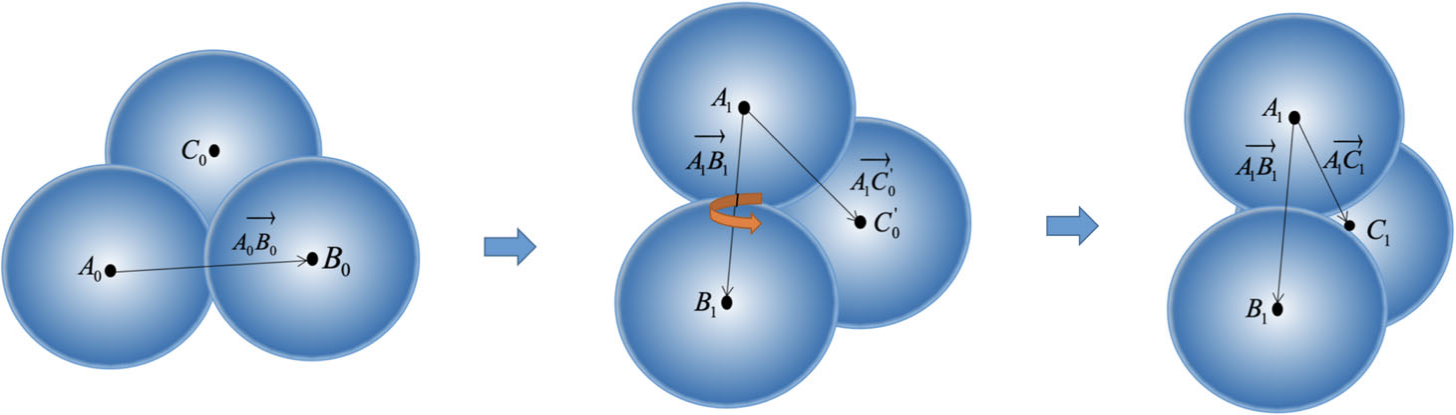
The rotation angle of a macromolecule. The left subfigure is the initial status, and the right subfigure shows the macromolecule *P* after rotating. The middle one is the intermediate rotation state which is only used to calculate the rotation matrix. Two linearly independent vectors can uniquely determine the angle of rotation of a macromolecule. First, the rotation angle of *A*_0_*B*_0_ to *A*_1_*B*_1_ is solved. The rotation of the macromolecule around the *A*_1_*B*_1_ axis is then determined by the vector *A*_1_*C*^′^_0_ and *A*_1_*C*^′^_1_. The rotation angle is obtained by superimposing the above two angles. In our simulation, since the overall size of the macromolecule is mostly concentrated around 120, the diameter of filaments and membranes is set to 40, and 80 respectively

As shown in Fig. 5, macromolecule *P* is rotated from (*P*_0_) to subfigure *P*_1_ during time *δt*. Three points *A*_0_,*B*_0_,*C*_0_ move to the point *A*_1_,*B*_1_,*C*_1_ via the above transformation. The rotation matrix *R*_*AB*_ of *A*_0_*B*_0_ to *A*_1_*B*_1_ can be obtained, which represents the rotation of macromolecule *P* except the rotation with *AB* as an axis. To solve the rotation with *AB* as the axis, it is necessary to consider the motion of point *C*_0_. The intermediate vector *A*_1_*C*^′^_0_ is solved by *A*_0_*C*_0_ and *R*_*AB*_, then the second rotation matrix *R*_*AC*_ of *A*_1_*C*^′^_0_ to *A*_1_*C*_1_ is obtained. The final rotation matrix *R*_*P*_ is calculated by superimposing *R*_*AB*_ and *R*_*AC*_, which is the rotation matrix of macromolecule *P*.

For each complex, density maps are generated at 4 nm resolution and with pixel size of 1 nm using the PDB2VOL program of the Situs 2.0 package [32].Then based on the coordinate,orientation and the density map of single macromolecule, the overall density map could be generated. As for a deformable structure, it density map is generated based on the final shape after packing, and the rotation angle is set to zero.

## Experiment of packing subcelluar structures together and generating simulated cryo-electron tomogram

In order to verify the effectiveness of our method, four experiments with different type of Macromolecules and ultrastructure are carried out. The first one is a packing of 400 macromolecules with 20 types which is used to compare the crowded level of our method and single-ball method. The second one is the packing of five macromolecules to show the process and result clearly. The third one is the packing of six macromolecules, one filament and one membrane which is used to show the ability of packing deformable and undeformable sturcture under a unified framework. The fourth experiment shows the deformation process of a single deformable structure. All the rendering result is visualized by Visual Molecular Dynamics(VMD) [33]. The PDB view of all experiments are visualized by Chimera [34]. The video of the above experiments could be found in the Additional file 2.

### Crowded packing of macromolecules

**Fig. 6:**
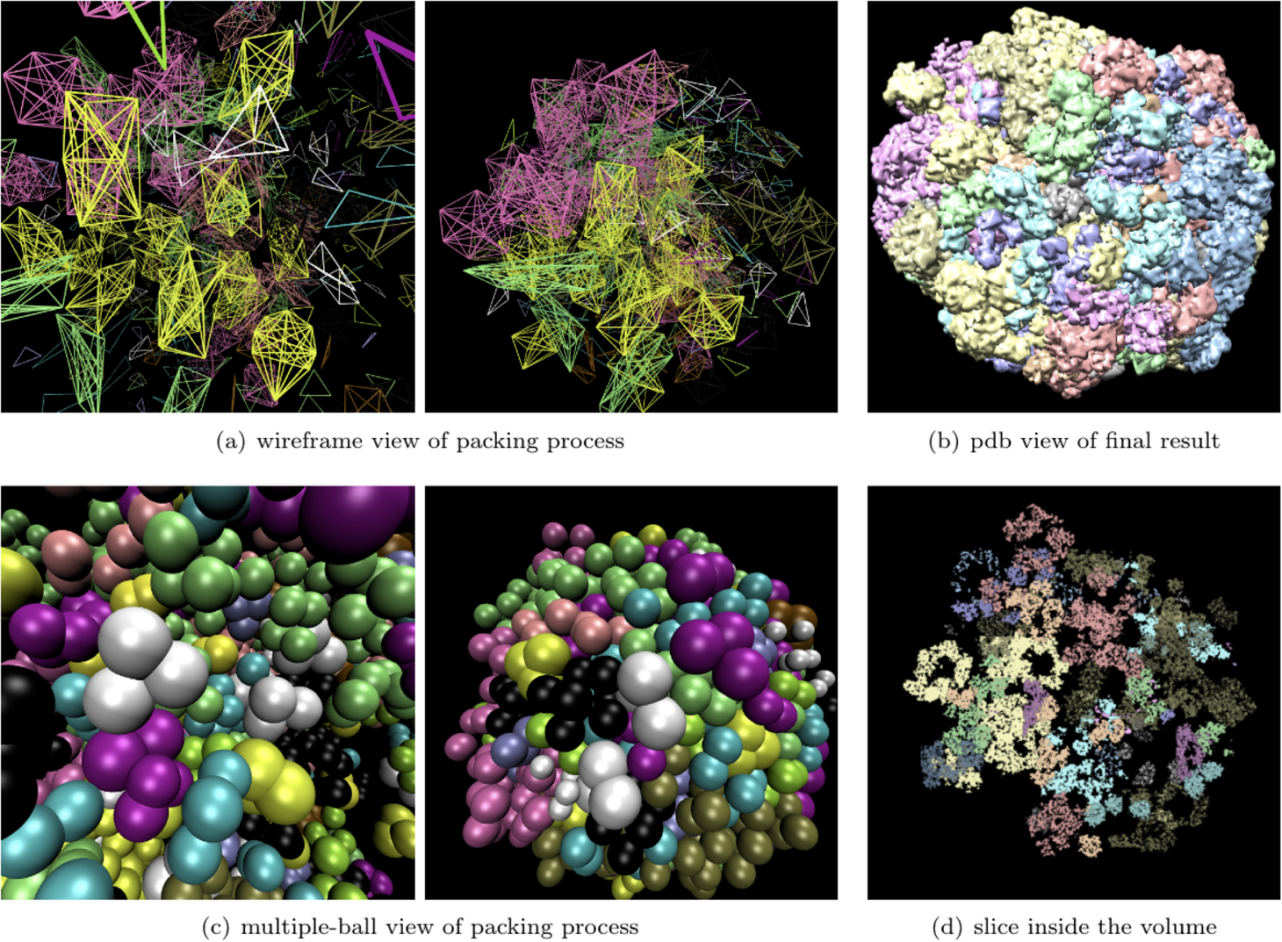
Packing process of 400 macromolecules with 20 types. Subfigure (a) and (c) shows wireframe view multiple-ball view of the packing process, respectively. In subfigure (**a**), each fully-linked body represents a macromolecule. The chemical bonds belong to the same macromolecule are marked in the same color. In subfigure (**c**), each macromolecule is represented by a cluster of balls in the same color. The macromolecules are initialized randomly in the scene, and they are far from each other. After packing, all macromolecules are brought together to form a tight cluster. Subfigure (**b**) is the 3D visualization of final PDB file, 20 types of macromolecules are marked in different colors. Subfigure (**d**) is a slice in this simulated volume

**Fig. 7:**
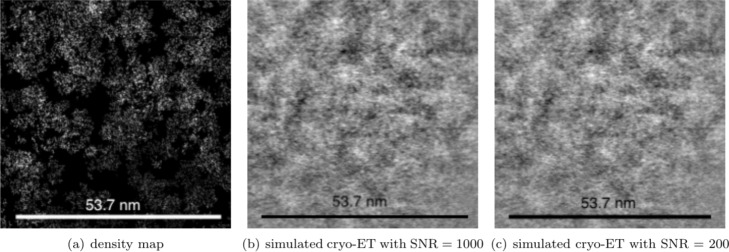
The density map and simulated cryo-electron tomogram of 400 packed macromolecules. Subfigure (**a**)shows the real states of packing result by a density map. Subfigure (**b**) and (**c**) are the simulated cryo-electron tomogram images under 1000 and 200 Signal-to-Noise-Ratio (SNR), respectively

**Packing of 400 macromolecules.** As shown in Fig. 6, 20×20*types*=400 macromolecules are packed in a box of 120*nm*×120*nm*×120*nm*. It could be seen in Subfig. 6a and Fig. 6c, all macromolecules gradually squeeze together from the initial dispersed state, forming a sphere-like structure under a force pointing to the scene center. Subfigure 6b shows the PDB view of the final result with different types of macromolecules in different colors. A slice in the volume is also shown in Subfig. 6d. In Fig. 7, the simulated cryo-electron tomography of this packing result is shown. In this experiment, macromolecules are tightly packed together, and the gaps between each other are very small.

Including 1A1F shown in Fig. 1, 20 kinds of different macromolecules which are varying in structure, size and shape were selected and approximated with multiple balls. In order to model them more reasonable, the number of balls was determined by macromolecule’s size using Eqs. 4 and 3. Atoms in a specific macromolecule are divided into *k* clusters according to their coordinates. The radius of each cluster is determined by the most distant atom in this cluster to the cluster center.

In a macromolecule, each pair of balls are linked by a chemical bond to form a stable topology. The length stretching and angular stretching of a chemical bond are constrained by the bonding potential function. Due to the high spring constant of the chemical bond, the relative position of balls in a macromolecule is hard to change, and the stability of the structure can be ensured.

The PDB view, clustering results, coarse-grained multiple-ball model, ball stick represent of 20 selected macromolecules are shown in Figs. S1, S2, S3, S4 in supplementary document, respectively.

**Fig. 8:**
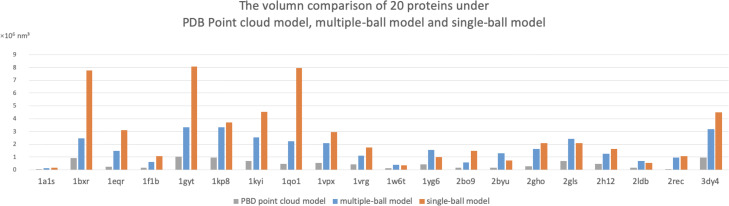
The volume comparison of the isosurface of PDB point cloud model, our multiple-ball model and single-ball model. The point cloud isodurface could be regarded as the true volume of a macromolecule. The calculation method is illustrated in supplementary Document section S3. This result shows that our multiple-ball model has a rather small volume the single-ball model. For 75% macromolecules, our model can save space for 11%-72% comparing with the single-ball model, especially 1BXR, 1GYT and 1QO1

**Volume comparison.** As shown in Fig. 8, the PDB point cloud model isosurface volume, multi-ball model volume and single-ball model volume of the 20 macromolecule models were determined. The volume of the PDB point cloud isosurface is the smallest one, which can be regarded as the true volume of each macromolecule. The atoms in a macromolecule form a point cloud, and the isosurface of the point cloud of all 20 macromolecules are shown in Fig. S5 in supplementary document. The calculation method is illustrated in supplementary document (Section S3) as well. Compared to the single-ball model, there is much less empty space in the multi-ball model, which means it is easier to achieve a tightly packed result. For 75% macromolecule, the multi-ball model is able to save 11%-72% volume, and our model has obvious advantages over the single-ball model in modeling macromolecule 1BXR, 1GYT and 1QO1. However, for some special macromolecules, our method does not show an advantage, which is also our follow-up research.

**Fig. 9:**
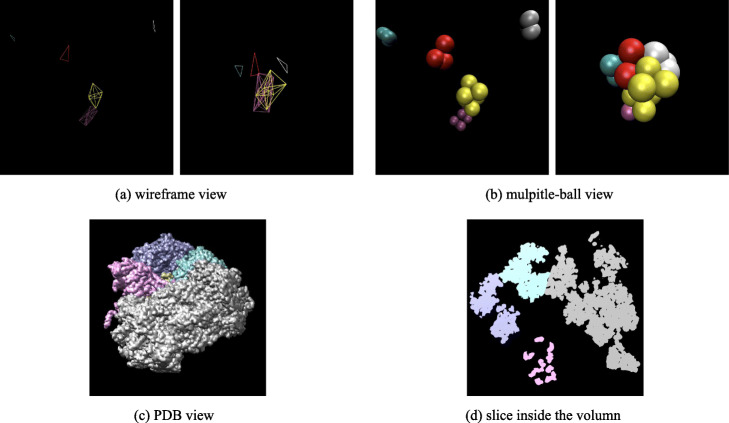
The packing process of 5 macromolecules. To show the packing process clearly, a small scene with only five macromolecules (1KY1, 1VPX, 1W6T, 2BO9, 2GHO) is set. Subfigure (**a**) and (**b**) are the wireframe view and multiple-ball view of the packing process, respectively. It could be seen clearly that five macromolecules moving toward each other and finally squeeze together. Subfigure (**c**) is the PDB view of the final status. Subfigure (**d**) shows the slices inside the volume

**Fig. 10:**
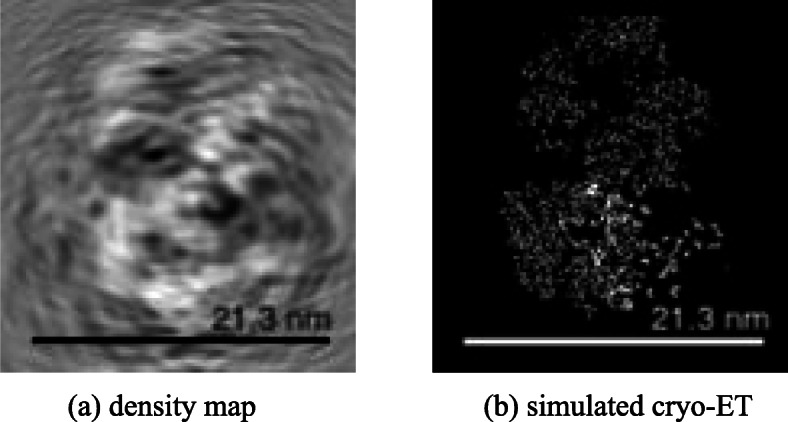
The density-map and simulated cryo-electron tomogram of 5 macromolecules (1KY1, 1VPX, 1W6T, 2BO9, 2GHO). Subfigure (**a**) is the selected density map slice of the packing volume corresponding to Fig. 9. Subfigure (**b**) is the simulated cryo-ET image with *SNR*=1000

**Small scene with 5 macromolecules.** To show the packing process clearly, an experiment with only five macromolecules is conducted. As shown in Fig. 9, five macromolecules (1KY1, 1VPX, 1W6T, 2BO9, 2GHO) are put in the scene with 50*nm*×50*nm*×50*nm*. The wireframe view (see Fig. 9a) and the multiple-ball view (see Fig. 9b) display the packing process clearly. Five macromolecules are moving from distance to center and eventually squeeze together. The final PDB view (see Fig. 9c) and inside slice (see Fig. 9d) demonstrate that the macromolecules are indeed tightly packed. The density map and Cryo-electron tomogram 2D slice image corresponding to this packing result are shown in Fig. 10.

### Unified packing of non-deformable macromolecules and deformable ultrastructure

**Fig. 11:**
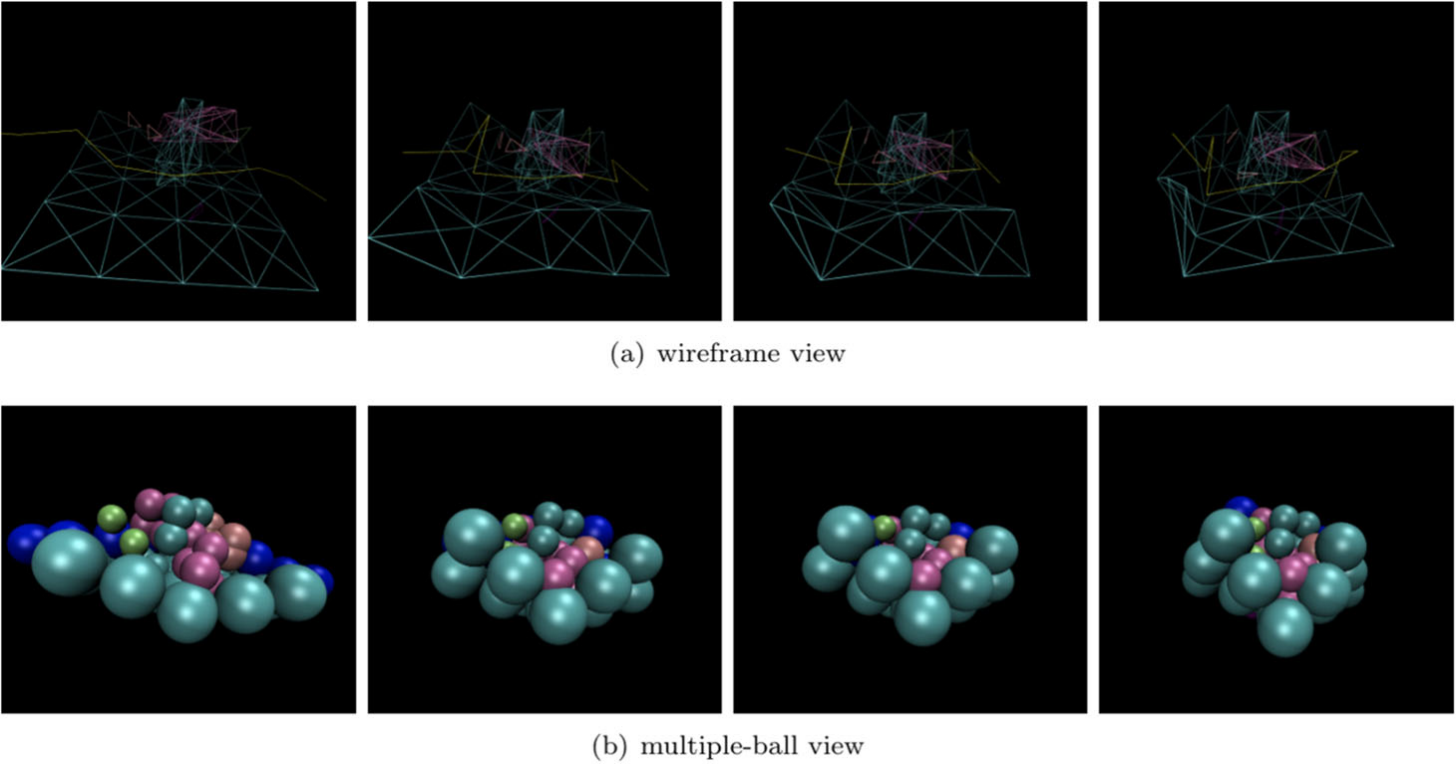
The packing process of six macromolecules, one filament and one membrane. There are six kinds of macromolecules in this scene: 1A1S,1BXR,1EQR,1F1B and 1GYT. During this process, macromolecules are moving toward each other and the membrane and filament are deforming. Subfigure (**a**) is the wireframe view of the packing process, in which the membrane is represented by sky blue lines, the filament is represented by yellow lines. Subfigure (**b**) is the multiple-ball view with membrane marked by sky blue and filament marked by bright blue

**Fig. 12:**
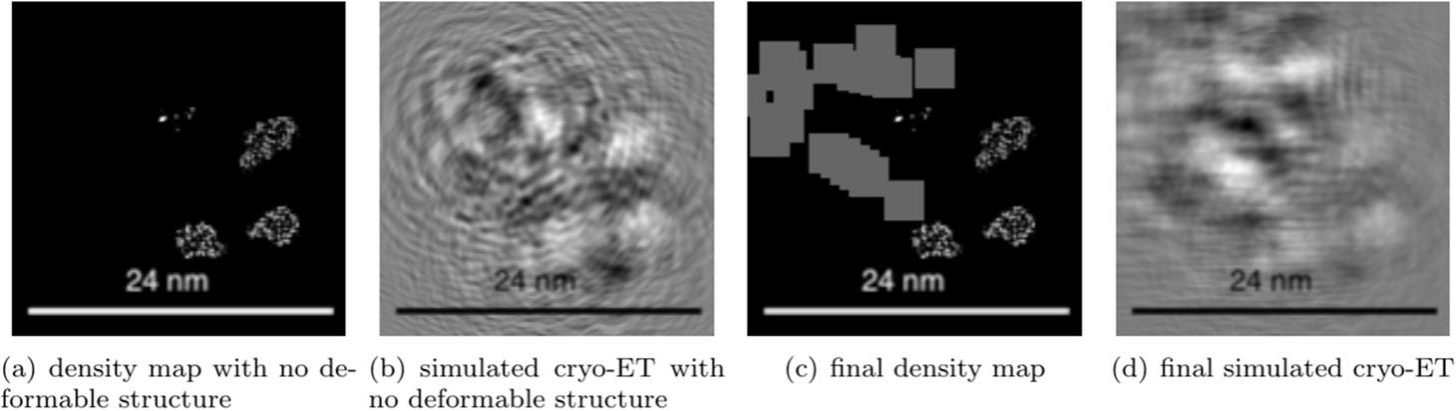
The density map and simulated Cryo-electron tomogram of six macromolecules, one filament and one membrane. A membrane is recorded in selected slice. Subfigure (**a**) is the density map without membrane. In this slice, three macromolecules could be seen. Subfigure (**b**) is the simulated Cryo-electron tomogram 2D slice corresponding to (a). Subfigure (**c**) is the final density map with a membrane in it, the membrane is shown in dark grey. Subfigure (**d**) is the final simulated cryo-electron tomogram with a membrane in it

**Unified packing of non-deformable and deformable structures.** Our method can simulate non-deformable structures (macromolecules) and deformable structures (filaments, membranes) under a unified framework. In order to show the simulation results clearly, a small scene packing process including six macromolecules, a filament, and a membrane was performed. The names of the selected macromolecules are 1A1S, 1BXR, 1EQR, 1F1B and 1GYT. As shown in Fig. 11, the wireframe view and the multi-sphere view of the packing process are shown separately. It can be clearly seen that under the external forces, structures are gathering toward the center, and the membrane (sky blue wireframe and small ball) and the filament (bright blue wireframe and small ball) are deforming. The corresponding density map and the cryo-electron tomography are shown in Fig. 12.

For single-ball model, since the size of the minimum bounding ball changes as these structures deforming, it is not able to reasonably simulate the filament and membrane structures with deformable properties. In contrast, our method represent the deformation of filaments and membranes by changing the relative position between multiple balls. The deformable topology of filament and membrane are shown in Fig. 2, this structure ensures their flexibility.

**Fig. 13:**
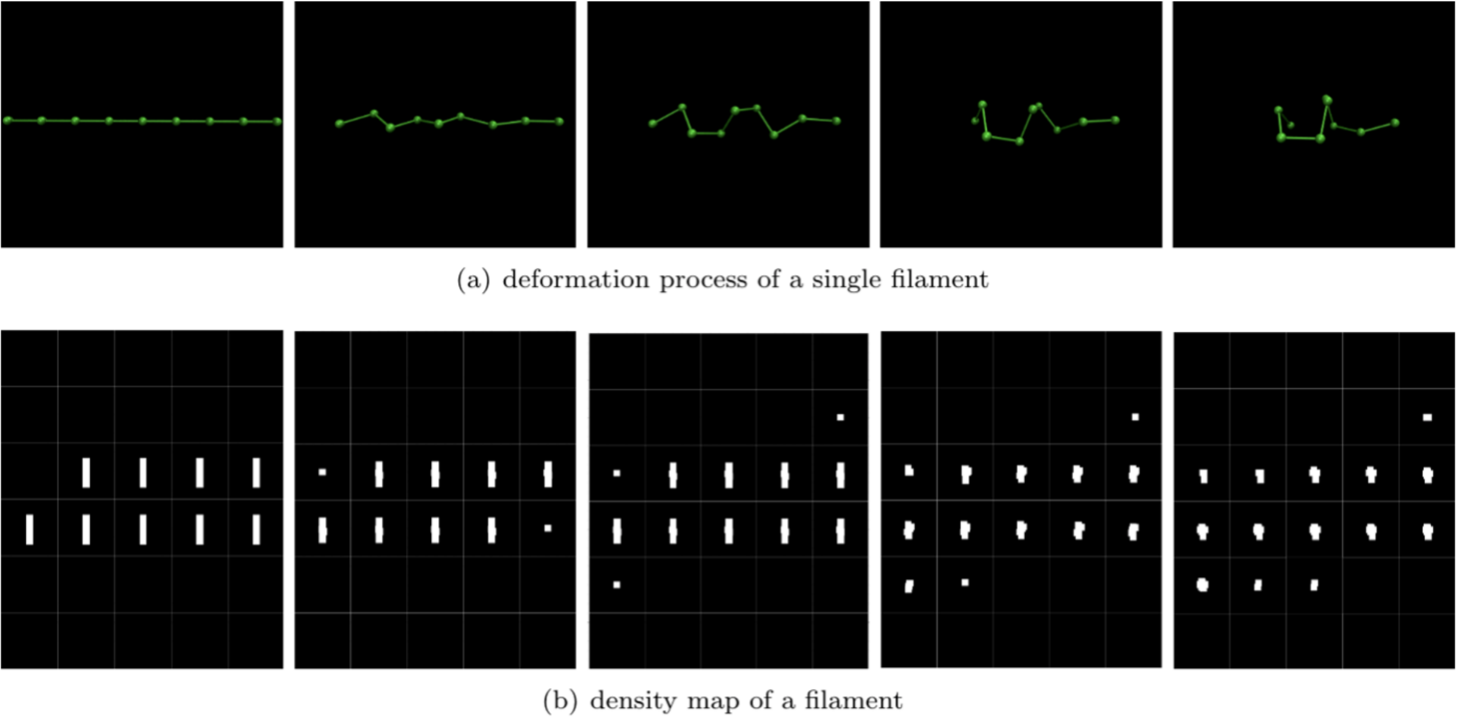
The deformation of a filament. In this figure, a single filament deforms under force field. Subfigure (**a**) shows the 3D ball-stick view, and subfigure (**b**) shows the corresponding 2D density map slice with 36 pieces. During deformation, the angle between two bonds is flexible, so that the filament can curl effectively and change its shape. Limited by the chemical bonds’ length, the filament cannot be stretched

**Fig. 14:**
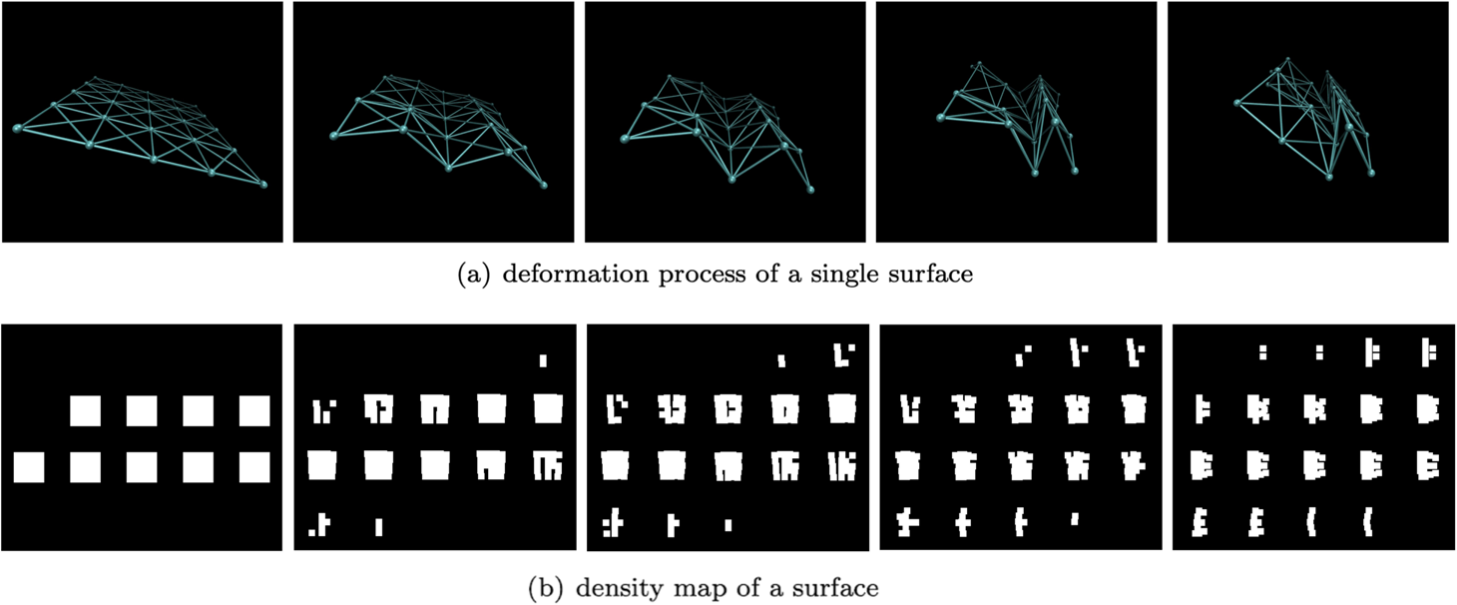
The deformation of a membrane. Subfigure (**a**) shows the deformation process of a single membrane under our force field in 3D ball-stick view. Subfigure (**b**) shows the corresponding 2D density map slices with 20 pieces each. During this process, a membrane is able to change its shape but cannot be stretched. Since a membrane has more bonds than a filament, it is a little constructed than filament

**Deformation of single deformable structure.**In order to verify the capability of the deformation process simulation, we simulated the deformation of a single filament and a single membrane. As shown in Fig. 13, a filament made up by 9 small balls is deforming under the applied force. The 3D deformation process is shown in Fig. 13b. In order to better show the shape of the filament during the deformation process, 36 slices corresponding to each state are generated (see Fig. 14). Figure 14 shows the deformation process of a single membrane, in which Fig. 14a and Fig. 14b are the 3D deformation process and the slice image, respectively. Due to having more chemical bonds, the membrane deforms much less than the filament.

## Image processing method testing

### Test of DoG reference free particle picking method

The first step to any analysis done on the cryo-ET is to identify the locations of existing particles and structures in the image. In the past, several machine learning algorithms have been proposed for reliable and efficient particle picking in a cryo-ET, but the performance of such methods still remain unconvincing due to the lack of labelled data for testing. The simulated tomograms can serve as the ground truth for this exact purpose.

Specifically our study focused on the performance of the Difference of Gaussian (DoG) reference free particle picking method. The method generates two different Gaussian filtered images and take the subtraction of the two. Then predicted locations of potential particles are then determined by detecting peaks in the resulting map.

The Gaussian filtered image is obtained using the Gaussian function *G*(*σ*) for a given *σ* value. More specifically, the filtered image *I*_*G*_(*σ*) is obtained by multiplying the original image and the Gaussian function $G(\sigma) =\frac {1}{\sigma \sqrt {2\pi }}e^{-\frac {r^{2}}{2\sigma ^{2}}}$. The difference of Gaussian image transformation is obtained by subtracting two such filtered images using two different *σ*, hence the name DoG. Typically, the ratio of the two *σ* is set to 1.1 for cryo-ET.

After the DoG image transformation is generated, local density peaks are detected to identify the possible particle locations. Such local peaks are filtered using a density threshold *T* to remove those resulted by noise.

$$\begin{array}{*{20}l} T = m + t \cdot \frac{M - m}{20} \end{array} $$

where M is the maximum density value of all local peaks, m is the minimum density value of all local peaks, and *t* is the threshold level at which we wish to filter the noise. The threshold level *t* is set to 5 for this particular performance evaluation.

#### Evaluating the performance of particle picking

To evaluate the Difference of Gaussian method mentioned above, there need to be a metric to identify true positives along with other useful information such as false negatives and false positives, and this process requires knowledge of the exact molecules and the locations in the cryo-ET.

Fortunately, we could use the simulated tomograms as the ground truth for this task. The labels of the particles, their locations, angles, radius, and other information are generated and embedded within the simulation process itself, eliminating the need for human annotation or other predictive measures.

Knowing the correct location and radius information, we could determine the true positives if there exist a detected local density peak close to the center of the particle. In other words, given the center and the radius of a particular particle, we consider it to be accurately labelled if one density peak is detected within the radius of the particle.

Precision and recall are effective method for performance analysis, and F score is used for overall particle picking performance defined by

$$F-score = \frac{2\cdot precision \cdot recall }{precision + recall} $$

#### Test results of particle picking

Considering the following equations for precision and recall:

$$\begin{array}{*{20}l} precision &= \frac{TP}{TP + FP}\\ recall &= \frac{TP}{TP + FN} \end{array} $$

where *TP* is the number of true positives (see true positive definition above), *FP* is the number of false positives, and *FN* is the number of false negatives.

The simulated tomogram contained 400 particles which was then used as the ground truth, and the Difference of Gaussian particle picking method predicted potential locations for 400 particles. Note that *TP*+*FP* is the total number positive predictions the method make, which equals to 400, and *TP*+*FN* is the total number of true positives the tomogram had, which also equals to 400. Therefore, the precision and recall values would be exactly the same. Now consider the F-score, notice that the value of the F-score would also equal to the precision and recall. Below in the table, we only present the F-score value, but keep in mind that this would also be the precision and recall value.

Difference of Gaussian particle picking method at different *σ*_1_ value was performed on the simulated tomogram and the particle picking results were analyzed giving the F-score. The experiment result is shown in Table 1.

**Table 1:** Performance test of Different of Gaussian particle picking method at different scaling factors

	F-Score
*σ*_1_=2	0.08=32/400
*σ*_1_=3	0.08=32/400
*σ*_1_=4	0.0825=33/400
*σ*_1_=5	0.075=30/400
*σ*_1_=6	0.085=34/400
*σ*_1_=7	0.075=30/400

**Fig. 15:**
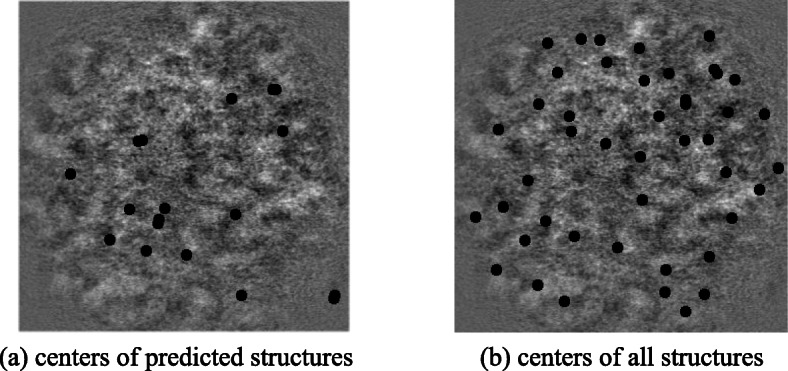
Ground truth and the result of DoG particle picking method. SubfIgure (**a**) shows the centers of actual particles in this tomogram where as subfigure (**b**) shows the centers of predicted particles picked by DoG algorithm

Figure 15 illustrates the simulated tomogram as well as the center of predicted particle locations given by the Difference of Gaussian particle picking method where the centers of the predictions are marked with black dots.

### Test of semantic segmentation method using fully convolutional network

After identifying locations of potential particles, another useful method to be applied for Cryo-ET analysis is semantic segmentation. Being able to decode the structural information stored in a tomogram requires divide the tomogram in separate pieces for each structure for better analysis. However, one problem that still exists in the research field of Cryo-ET semantic segmentation is the trade-off between model accuracy and the amount of required training data. Models with high accuracy are typically deep learning models that require a large amount of high quality labelled training data. And because of the challenging and time-demanding nature of image segmentation and labeling, such training data is very scarce. Other methods that do not have as high demand for training data usually do not have comparable results as the deep learning models. To resolve this issue, our simulated data can serve as the labelled training data for high performing deep learning models.

Fully Convolutional Network (FCN) is one of the most popular neural network model due to its ability to learn both higher level patterns and low level details. Specifically, the FCN will make predictions of a tomogram by analyzing every pixel. The network contains 3D convolutional layers to capture high-level patterns as well as upsampling and pooling layers for finer segmentation information. The exact architecture of the network is given in supplementary material Section S4 and Fig. S6. This encoder-decoder variant of the FCN adds skip connections in the upsampling phase that combines the higher layers with the lower pooling layers to fully utilize global context information while integrating the local information to accurately map low-resolution images to voxel-wise predictions. In a previously published paper, it is found that this model has consistently outperformed other similar variants of FCNs for the segmentation task [7]. Our simulation provides 3D simulated cryo-ET data that can serve as the training data for EDSSN3D network and reduce the amount of training data preparation required of this model and thus successfully break the originally thought trade-off between accuracy and data preparation.

#### Evaluation of image segmentation method

To evaluate this network model, the annotated data was split into two sets: 90 percent of the prepared data was used as the training data for the network, and the remaining 10 percent of data was held back for evaluation purposes. After the training is done, the 10 percent held out data would then be fed into the trained network and predictions would be generated with the real labels hidden from the network. The predictions would then be compared to the real label.

Pixel accuracy ${accuracy = \frac {TP}{\text {all data points}}}$ was calculated by counting the number of pixels the network made the correct predictions, where *TP* is the number of true positives. In our experiment, the final accuracy of this method is 0.7015. The simulation results produced by the method proposed in this paper pose a greater challenge to existing image segmentation methods. There is an urgent need to develop new algorithms to enable better experimental results.

## Discussion

In this paper, we proposed the first framework to generate realistic simulated cryo-electron tomograms including non-deformable macromolecular complexes and deformable ultrastructures under a crowded status. The simulated cryo-ET can be regarded as labeled data to assist sub-sequence analysis methods.

To achieve this goal, we approximated a macromolecules using multiple balls with fixed relative positions, and approximated ultrastructure (membranes, filaments) using multiple balls with flexible relative positions. Compared to the traditional method [18] in which each macromolecule is approximated by a single minimum boundary sphere, small square or small cylinder, our multi-ball model can achieve a higher spatial compression ratio. We selected 20 representative macromolecules for experiments, taking 5000 atoms as a unit and dividing the atoms into near clusters based on coordinates. Taking the cluster center as the ball’s center, the distance of the farthest atom in this cluster as the ball’s radius, a multi-sphere model is obtained. Compared to the single-ball model, our method reduces the volume by 11%-72%.

Besides, it is difficult for the single-ball model to dynamically represent the change of minimum bounding sphere size due to the shape change, while our multiple-ball model can effectively express the deformability of the structure through the relative displacement of the inner balls. In this paper, different typologies are designed to describe macromolecules, filaments and membrane with different deformability. In macromolecule, all balls are chemically linked. In the initial model of filament, the balls are col-linear and are connected end to end in order. Balls in the initial model of the membrane are co-planar and are connected to their neighbors in the eight directions of up, down, left, right and four diagonal directions. For macromolecules, the topology determines that the structure does not deform as long as the bond length does not change. For filaments and membranes, the topology determines that they can deform flexibly. Compared to the single-ball model, the bonding force is need to be considered in addition to the non-bonding force and external force. The elasticity of the bond length determines its stretching capability, and the angle elasticity determines its bending capability. A single filament and membrane simulation experiment proves that the multi-ball model can effectively represent the deformation process of the structure in space. For the multi-ball model, since one macromolecular complex or ultrastructure is described by multiple points, the rotation angle during motion is achievable. In contrast, traditional single ball model does not require this step and the angle of rotation is randomly set which not fit the physical law. In this paper, the rotation angle of a macromolecule is uniquely determined by three non-col-linear points in it, which is why the minimum cluster number is set to 3.

In this paper, six non-deformable macromolecules and two deformable filaments and membranes are placed in the same scene, and the object with different propertied could be distinguished easily. Experiments show that the proposed method can obtain realistic and highly crowded simulated cryo-electron tomogram, including deformable objects and non-deformable objects.

Our simulation data can be used to evaluate existing image processing methods. Machine learning methods can deliver high accuracy in various tasks, sometimes outperforms humans. One major shortcomings of machine learning and deep learning methods is the need for large quantity of accurately labelled data. In the field of Cryo-ET analysis, the lack of labelled data has resulted in unconvincing and sometimes inconclusive algorithm searching. By generating realistic simulated tomograms, we can bridge the gap by providing benchmarks for various algorithms [35–40] for cryo-ET analysis.

The accuracy and performance of Difference of Gaussian particle picking method is highly dependent on the hyperparameter *σ*_1_. With real tomograms, the performance evaluation can be quite challenging and error prune as the particles in the image needs to be manually identified. In a simulated image, the exact locations of every particle are known, allowing the true precision and recall to be calculated. Simulated tomograms gives more accurate evaluation schemes. For supervised deep learning methods, the availability of correctly labelled data is even more important. By using this simulation framework, we can generate large quantities of tomograms with the correct label which can then serve as the training and testing data. The Cryo-ET data generated by our method is closer to the real state, so that its analysis is more difficult than previous simulated cryo-ET. The existing image processing methods still have much room for improvement on our simulation data, and new algorithms are expected to be proposed to obtain better experimental results.

## Conclusions

In this paper, we proposed a unified framework to simulate cryo-ET with both deformable and non-deformable subcelluar structures, including macromolecules, filaments and mambranes. Our multiple-ball model makes it possible to achieve more crowded status than sinlge-ball model, which is more close to real status in cell. The proposed packing algorithm is combined with the Molecule Dynamics, which makes the simulated cryo-ET results more reliable. The experiment results shows that our method can pack subcelluar structures more tightly. The simulated cryo-ET data is easy to be labeled and able to improve DoG particle picking method and bioimage processing methods based on Machine Learning.

In the future, we will generate cryo-ET datasets using this method to provide help of all the researchers in this research field. At the same time, we will focus on improving the Molecule Dynamics model to make it more accurate. Futhermore, We will try to improve the density map merging process of deformable structures and nondeformable structures to make the simulated result clearer.

## Supplementary information

**Additional file 1** Supplementary document. Section S1: The visualization of 20 selected macromolecules. Section S2: K-means method. Section S3: The calculation of PDB volume. Section S4: The network architecture of EDSSN3D.

**Additional file 2** A video of all the experiments in this paper, including the visualization of the packing process, the simulated density map and the simulated cryo-ET.

## Data Availability

The macromolecular complexes can be found at the Protein Data Bank (the Research Collaboratory for Structural Bioinformatics: http://www.rcsb.org/pdb).

## Abbreviations

cryo-ET: Cryo-electron tomography
PDB: Protein data bank
SNR: Signal to noise ratio

## References

[1] Irobalieva RN, Martins B, Medalia O (2016). Cellular structural biology as revealed by cryo-electron tomography. J Cell Sci.

[2] Fernandez-Leiro R, Scheres SH (2016). Unravelling biological macromolecules with cryo-electron microscopy. Nature.

[3] Cheng Y, Grigorieff N, Penczek PA, Walz T (2015). A primer to single-particle cryo-electron microscopy. Cell.

[4] Oikonomou CM, Chang Y-W, Jensen GJ (2016). A new view into prokaryotic cell biology from electron cryotomography. Nat Rev Microbiol.

[5] Wang F, Gong H, Liu G, Li M, Yan C, Xia T, Li X, Zeng J (2016). DeepPicker: a deep learning approach for fully automated particle picking in cryo-EM. J Struct Biol.

[6] Frank J (2017). Advances in the field of single-particle cryo-electron microscopy over the last decade. Nat Protoc.

[7] Liu C, Zeng X, Lin R, Liang X, Freyberg Z, Xing E, Xu M. Deep learning based supervised semantic segmentation of electron cryo-subtomograms. In: 2018 25th IEEE International Conference on Image Processing (ICIP). IEEE: 2018. p. 1578–82. 10.1109/icip.2018.8451386 PMC10552869 37799820

[8] Guay MD, Emam ZA, Anderson AB, Leapman RD (2019). Transfer learning for efficient segmentation of subcellular structures in 3-D electron microscopy. Biophys J.

[9] Li R, Zeng X, Sigmund SE, Lin R, Zhou B, Liu C, Wang K, Jiang R, Freyberg Z, Lv H, Xu M (2019). Automatic localization and identification of mitochondria in cellular electron cryo-tomography using faster-RCNN. BMC Bioinformatics.

[10] Kmiecik S, Gront D, Kolinski M, Wieteska L, Dawid AE, Kolinski A (2016). Coarse-grained protein models and their applications. Chem Rev.

[11] de Jong DH, Singh G, Bennett WD, Arnarez C, Wassenaar TA, Schafer LV, Periole X, Tieleman DP, Marrink SJ (2012). Improved parameters for the martini coarse-grained protein force field. J Chem Theory Comput.

[12] Bond PJ, Holyoake J, Ivetac A, Khalid S, Sansom MS (2007). Coarse-grained molecular dynamics simulations of membrane proteins and peptides. J Struct Biol.

[13] Tozzini V (2005). Coarse-grained models for proteins. Curr Opin Struct Biol.

[14] Al Nasr K, Chen L, Si D, Ranjan D, Zubair M, He J. Building the initial chain of the proteins through de novo modeling of the cryo-electron microscopy volume data at the medium resolutions. In: Proceedings of the ACM Conference on Bioinformatics, Computational Biology and Biomedicine. ACM: 2012. p. 490–7.

[15] Pierce BG, Hourai Y, Weng Z (2011). Accelerating protein docking in ZDOCK using an advanced 3D convolution library. PloS ONE.

[16] Lindow N, Baum D, Bondar A-N, Hege H-C (2013). Exploring cavity dynamics in biomolecular systems. BMC Bioinformatics.

[17] Ellis RJ (2001). Macromolecular crowding: an important but neglected aspect of the intracellular environment. Curr Opin Struct Biol.

[18] Pei L, Xu M, Frazier Z, Alber F (2016). Simulating cryo electron tomograms of crowded cell cytoplasm for assessment of automated particle picking. BMC Bioinformatics.

[19] Liu S, Wang B, Ban X. Multiple-scale simulation method for liquid with trapped air under particle-based framework. In: 2020 IEEE Conference on Virtual Reality and 3D User Interfaces (VR). IEEE: 2020. p. 842–50.

[20] Liu S, Wang X, Ban X, Xu Y, Zhou J, Zhang Y. Viscosity-based vorticity correction for turbulent sph fluids. In: 2019 IEEE Conference on Virtual Reality and 3D User Interfaces (VR). IEEE: 2019. p. 1048–9.

[21] Wang X, Liu S, Ban X, Xu Y, Zhou J, Wang C. Recovering turbulence details using velocity correction for sph fluids. In: SIGGRAPH Asia 2019 Technical Briefs: 2019. p. 95–8.

[22] Peng K, Zheng L, Xu X, Lin T, Leung VC. Balanced iterative reducing and clustering using hierarchies with principal component analysis (PBIRCH) for intrusion detection over big data in mobile cloud environment. In: International Conference on Security, Privacy and Anonymity in Computation, Communication and Storage. Springer: 2018. p. 166–77.

[23] Lahari K, Murty MR, Satapathy SC. Partition based clustering using genetic algorithm and teaching learning based optimization: performance analysis. In: Emerging ICT for Bridging the Future-Proceedings of the 49th Annual Convention of the Computer Society of India CSI Volume 2. Springer: 2015. p. 191–200.

[24] Kriegel H-P, Kröger P, Sander J, Zimek A (2011). Density-based clustering. Wiley Interdiscip Rev Data Min Knowl Disc.

[25] Amini A, Wah TY, Saybani MR, Yazdi SRAS. A study of density-grid based clustering algorithms on data streams. In: 2011 Eighth International Conference on Fuzzy Systems and Knowledge Discovery (FSKD). IEEE: 2011. p. 1652–6.

[26] McNicholas PD (2016). Model-based clustering. J Classif.

[27] Mohri M, Rostamizadeh A, Talwalkar A. Foundations of Machine Learning: MIT press; 2018.

[28] Berman HM, Westbrook J, Feng Z, Gilliland G, Bhat TN, Weissig H, Shindyalov IN, Bourne PE (2000). The protein data bank. Nucleic Acids Res.

[29] Pedregosa F, Varoquaux G, Gramfort A, Michel V, Thirion B, Grisel O, Blondel M, Prettenhofer P, Weiss R, Dubourg V, Vanderplas J, Passos A, Cournapeau D, Brucher M, Perrot M, Duchesnay E (2011). Scikit-learn: machine learning in Python. J Mach Learn Res.

[30] Phillips JC, Braun R, Wang W, Gumbart J, Tajkhorshid E, Villa E, Chipot C, Skeel RD, Kale L, Schulten K (2005). Scalable molecular dynamics with NAMD. J Comput Chem.

[31] Slabaugh GG (1999). Computing euler angles from a rotation matrix. Retrieved on August.

[32] Wriggers W, Milligan R, McCammon J. Situs: A package for the docking of protein crystal structures to low-resolution maps from electron microscopy. In: BIOPHYSICAL JOURNAL. BIOPHYSICAL SOCIETY 9650 ROCKVILLE PIKE, BETHESDA, MD 20814-3998 USA: 1999. p. 23.

[33] Humphrey W, Dalke A, Schulten K (1996). VMD: visual molecular dynamics. J Mol Graph.

[34] Pettersen EF, Goddard TD, Huang CC, Couch GS, Greenblatt DM, Meng EC, Ferrin TE (2004). UCSF Chimera—a visualization system for exploratory research and analysis. J Comput Chem.

[35] Xu M, Singla J, Tocheva EI, Chang Y-W, Stevens RC, Jensen GJ, Alber F (2019). De novo structural pattern mining in cellular electron cryotomograms. Structure.

[36] Zeng X, Leung MR, Zeev-Ben-Mordehai T, Xu M (2018). A convolutional autoencoder approach for mining features in cellular electron cryo-tomograms and weakly supervised coarse segmentation. J Struct Biol.

[37] Zeng X, Xu M. Gum-Net: Unsupervised geometric matching for fast and accurate 3D subtomogram image alignment and averaging. In: Proceedings of the IEEE/CVF Conference on Computer Vision and Pattern Recognition: 2020. p. 4073–84. 10.1109/cvpr42600.2020.00413 PMC7955792 33716478

[38] Zeng X, Xu M. AITom: Open-source AI platform for cryo-electron Tomography data analysis. arXiv preprint arXiv:1911.03044. 2019.

[39] Hrabe T, Chen Y, Pfeffer S, Cuellar LK, Mangold A-V, Förster F (2012). PyTom: a python-based toolbox for localization of macromolecules in cryo-electron tomograms and subtomogram analysis. J Struct Biol.

[40] Moebel E, Martinez-Sanchez A, Lariviere D, Fourmentin E, Ortiz J, Baumeister W, Kervrann C. Deep learning improves macromolecules localization and identification in 3D cellular cryo-electron tomograms. bioRxiv. 2020.

