# Supplementary material for: A unified framework for packing deformable and non-deformable subcellular structures in crowded cryo-electron tomogram simulation
Source: BMC Bioinformatics. 2020 Sep 9;21:399. doi: 10.1186/s12859-020-03660-w (PMC7488303; doi:10.1186/s12859-020-03660-w)
Supplement: Supplementary file 1 — Additional file 1 Supplementary document. Section S1: The visualization of 20 selected macromolecules. Section S2: K-means method. Section S3: The calculation of PDB volume. Section S4: The network architecture of EDSSN3D. [file 12859_2020_3660_MOESM1_ESM.pdf]

A Unified Framework for Packing Deformable and Non-deformable Subcellular Structure in Crowded Cryo-electron Tomogram Simulation
# – Supplementary Document

Sinuo Liu[1,2], Xiaojuan Ban*[1], Xiangrui Zeng[2], Fengnian Zhao[3], Yuan Gao[2], Wenjie Wu[4], Hongpan Zhang[5,6], Feiyang Chen[7], Thomas Hall[2], Xin Gao[8], and Min Xu†[2]

[1]Beijing Advanced Innovation Center for Materials Genome Engineering, School of Computer and Communication Engineering, University of Science and Technology Beijing, Beijing, 100083, China
[2]Computational Biology Department, School of Computer Science, Carnegie Mellon University, Pittsburgh, 15213, USA
[3]School of Computer Science, Beijing University of Posts and Telecommunications, Beijing, China
[4]School of Mechanical, Electrical and Information Engineering, Shandong University, Shandong, China
[5]WuYuzhang Honors College, Sichuan University, Sichuan, China
[6]College of Life Science, Sichuan University, Sichuan, China
[7]School of Information Science and Technology, Beijing Forestry University, Beijing, China
[8]King Abdullah University of Science and Technology (KAUST), Computational Bioscience Research Center (CBRC), Computer, Electrical and Mathematical Sciences and Engineering (CEMSE) Division, Thuwal, Saudi Arabia

S1 The Visualization of 20 Selected Macromolecule

The classical PDB multi-ball view, clustering results, coarse-grained multiple-ball model, coarse-grained ball-stick represent of 20 selected macromolecules are shown in Figure S1, S2, S3, S4, respectively. The names of these macromolecules are 1A1S, 1BXR, 1EQR, 1F1B, 1GYT, 1KP8, 1KYI, 1QO1, 1VPX, 1VRG, 1W6T, 1YG6, 2BO9, 2BYU, 2GHO, 2GLS, 2H12, 2IDB, 2REC, 3DY4.

S2 K-means Method

The idea of the k-means algorithm is that for a given sample set, it is divided into k clusters according to the distance between the samples, making the points in the cluster as close as possible, and making the distance between the clusters as large as possible.

K-means algorithm is as follows [2]:

*Corresponding author email: banxj@ustb.edu.cn
†Corresponding author email: mxu1@cs.cmu.edu

Algorithm 1 k-means methods

Require: The input is the sample set $D = \{x_1, x_2, \ldots, x_m\}$, the cluster tree k, and the maximum number of iterations N.

Ensure: The output is cluster $C = \{C_1, C_2, \ldots, C_k\}$.

1. Randomly select k samples from dataset D as the initial k centroids: $\{\mu_1, \mu_2, \ldots, \mu_k\}$
2. For $n = 1, \ldots, N$:
   a. Initialize cluster C to $C_t = \emptyset$ in $t = 1, 2, \ldots, k$;
   b. For $i = 1, 2, \ldots, m$, calculate the distance between instance x_i and each centroid μ_i : $d_{ij} = \|x_i - \mu_j\|_2^2$, for each x_i find smallest d_{ij} in all d_{ij}, and put x_i in category λ_i, and then update $C_{\lambda_i} = C_{\lambda_i} \cup \{x_i\}$;
   c. For $j = 1, 2, \ldots, k$, recalculate a new centroid for all sample points in C_j: $\mu_j = \frac{1}{|C_j|} \sum_{x \in C_j} x$;
   d. If all k centroids have not changed, go to step 3.
3. Output cluster $C = \{C_1, C_2, \ldots, C_k\}$.

K-means clustering is one of the simplest and popular unsupervised machine learning algorithm [3], where there is no information about the correct outputs available at all, and the algorithm is left to spot some similarity between dierent inputs for itself. K-means has a great advantage in speed. Even if the clustering results of other algorithms are better than K-means, the speed is often slower than K-means. Moreover, K-means algorithm has only one super parameter K, so its robustness is very strong, unlike other K-means algorithm which is easy to be affected by other super parameters. To conclude, in most cases, K-means algorithm can achieve good results, and has advantages in time. K-means should be the first choice if it is not for the sake of high accuracy in a specific dataset.

S3 The Calculation of PDB Volume

The volume of each macromolecule in calcuated by two function in Matlab. First, the alphaShape of a macromolecule is generatied by the coordinate of all atoms. Then the volume of this isosurface could be calculated by the volume function.

alphaShape. An alphaShape creates a bounding area or volume that envelops a set of 2-D or 3-D points. Using alphaShape function [4] in Matlab can manipulate the alphaShape object to tighten or loosen the fit around the points to create a nonconvex region. After creating an alphaShape object, geometric queries can be performed. For example, it could be determined if a point is inside the shape, and the number of regions that make up the shape can be found. Useful quantities like area, perimeter, surface area, or volume can be calculated.

According to the Mathwork, alphaShape algorithm is as follows:

Algorithm 2 alphaShape

1. Starting from arbitrarily selecting a point P_1 in the point set P_S, the new point set P_{S2} consisting of points with a distance less than $2 \times \alpha$. And find the center P_{12} of points P_1 and P_2.
2. Traverse the point set P_{S2} and find the distance D from other points to P_{12} (except P_1 and P_2):
   a. If all the D obtained are greater than the radius α, and there are no other points in it, then it can be determined that the points P_1 and P_2 are edge contour points, and P_1 P_2 is the boundary line segment;
   b. If there is a value smaller than the radius α in D, and there are other points in the circle, you can judge that it is not an edge contour point, stop traversing, and go to step 3.
3. Select the next point in P_{S2} and follow steps 1. and 2. to judge until all points in P_{S2} are judged.
4. Take the next point in the P_S and follow the steps 1. 2. and 3. to judge until all points in the P_S are judged.

Volume. Volume function [5] is used to calculate the volume of 3D object. After using alphaShape function to find the boundary of each macromolecules, the volume function could be used to calculate the volume of the isosurface of each macromolecule.

S4 The Network Architecture of EDSSN3D

The network architecture of EDSSN3D [6] is shown in figure S6. A 3D encoder-decoder model for semantic segmentation of CECT. The input layer of the model expects 3D subtomograms of size $40 \times 40 \times 40$ voxels. The input layer is then connected to the encoder which is consisted of a series of fully convolutional and max pooling layers with ReLU activation. Then it is followed by a series of fully convolutional layers and upsampling layers (ReLU activation) with the same number of channels as the encoder. Skip connections were added to combine the higher layers with the lower pooling layers. For more information, please refer to papar [6].

References

[1] Eric F Pettersen, Thomas D Goddard, Conrad C Huang, Gregory S Couch, Daniel M Greenblatt, Elaine C Meng, and Thomas E Ferrin. Ucsf chimeraa visualization system for exploratory research and analysis. *Journal of computational chemistry*, 25(13):1605–1612, 2004.

[2] Stephen Marsland. *Machine learning: an algorithmic perspective*. Chapman and Hall/CRC, 2014.

[3] Jerome Friedman, Trevor Hastie, and Robert Tibshirani. *The elements of statistical learning*, volume 1. Springer series in statistics New York, 2001.

[4] Mathworkds. alphashape. `https://www.mathworks.com/help/matlab/ref/alphashape/`. Accessed on July 11, 2020.

[5] Mathworkds. volume. `https://www.mathworks.com/help/matlab/ref/alphashape.volume/`. Accessed on July 11, 2020.

[6] Chang Liu, Xiangrui Zeng, Ruogu Lin, Xiaodan Liang, Zachary Freyberg, Eric Xing, and Min Xu. Deep learning based supervised semantic segmentation of electron cryo-subtomograms. In *2018 25th IEEE International Conference on Image Processing (ICIP)*, pages 1578–1582. IEEE, 2018.

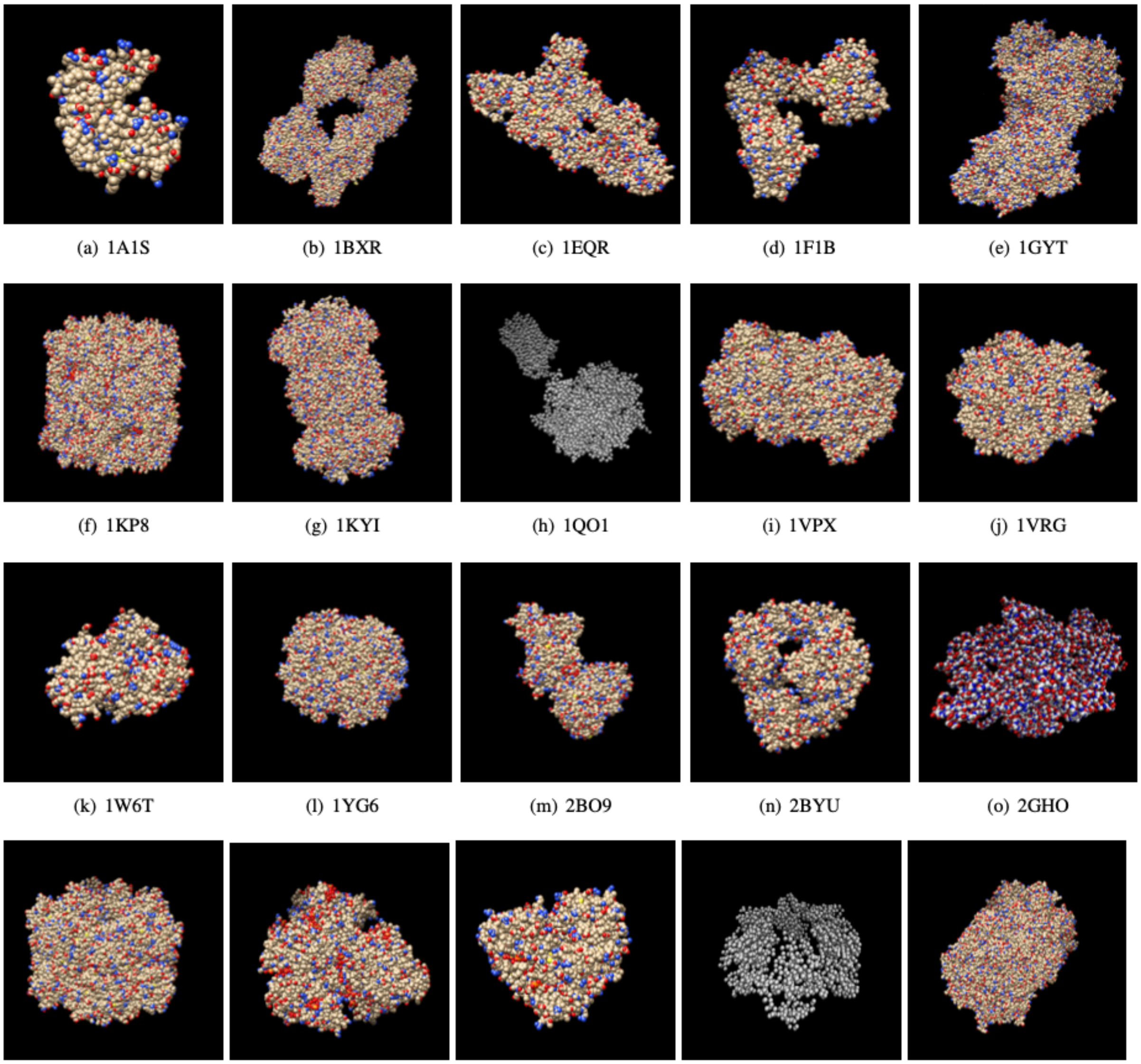

(a) 1A1S (b) 1BXR (c) 1EQR (d) 1F1B (e) 1GYT

(f) 1KP8 (g) 1KYI (h) 1QO1 (i) 1VPX (j) 1VRG

(k) 1W6T (l) 1YG6 (m) 2BO9 (n) 2BYU (o) 2GHO

(p) 2GLS (q) 2H12 (r) 2LDB (s) 2REC (t) 3DY4

Figure S1: The PDB view of 20 selected macromolecules. In this paper, 20 macromolecules are chosen to be packed in the experiment. The PDB view of these macromolecules are visualized by chimera [1] and shown in this figure.

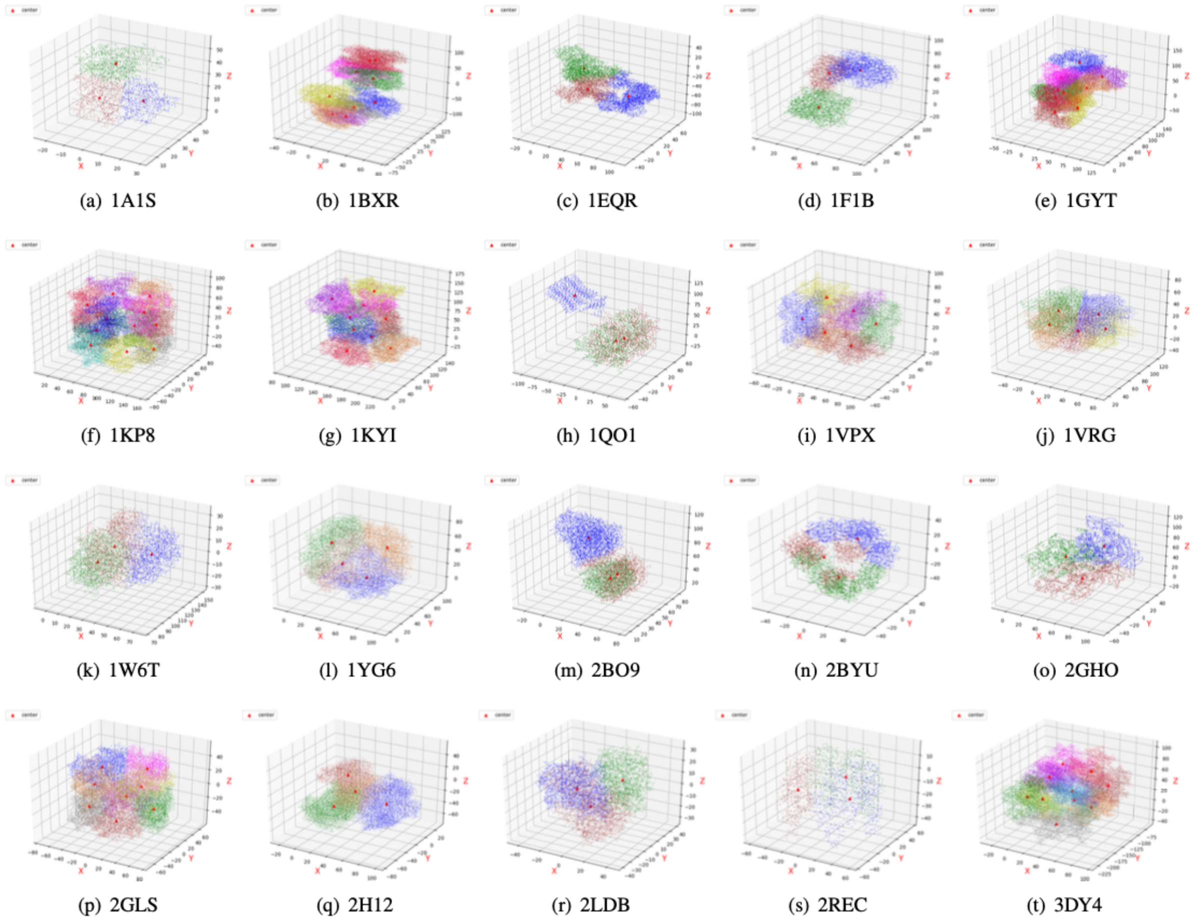

Figure S2: The clustering result of 20 macromolecules by k-means clustering method. Each macromolecule is divided into several clusters according to its atom number. The number of clusters is determined by equation 3 and 4. Atoms are divided into different clusters base on their distance away form these cluster centers. In this figure, the cluster centers are represented by red triangles, and atoms are represented by small points with different colors which means they are in different clusters.

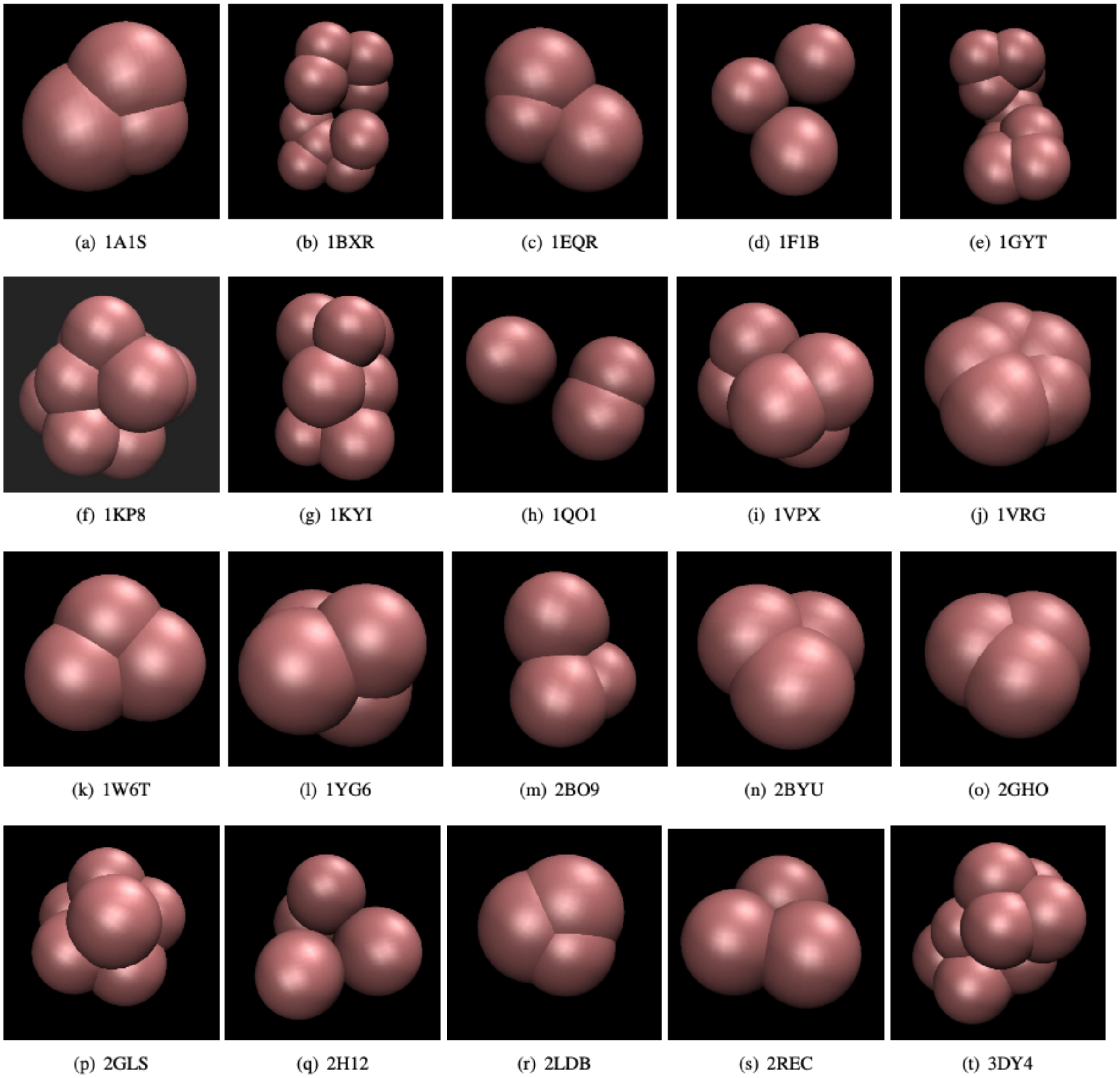

Figure S3: The 3D visualization of 20 macromolecules' coarse-grained multi-ball model. Each macromolecule is approximated by multiple balls. The number of balls is proportional to macromolecule's atom number.

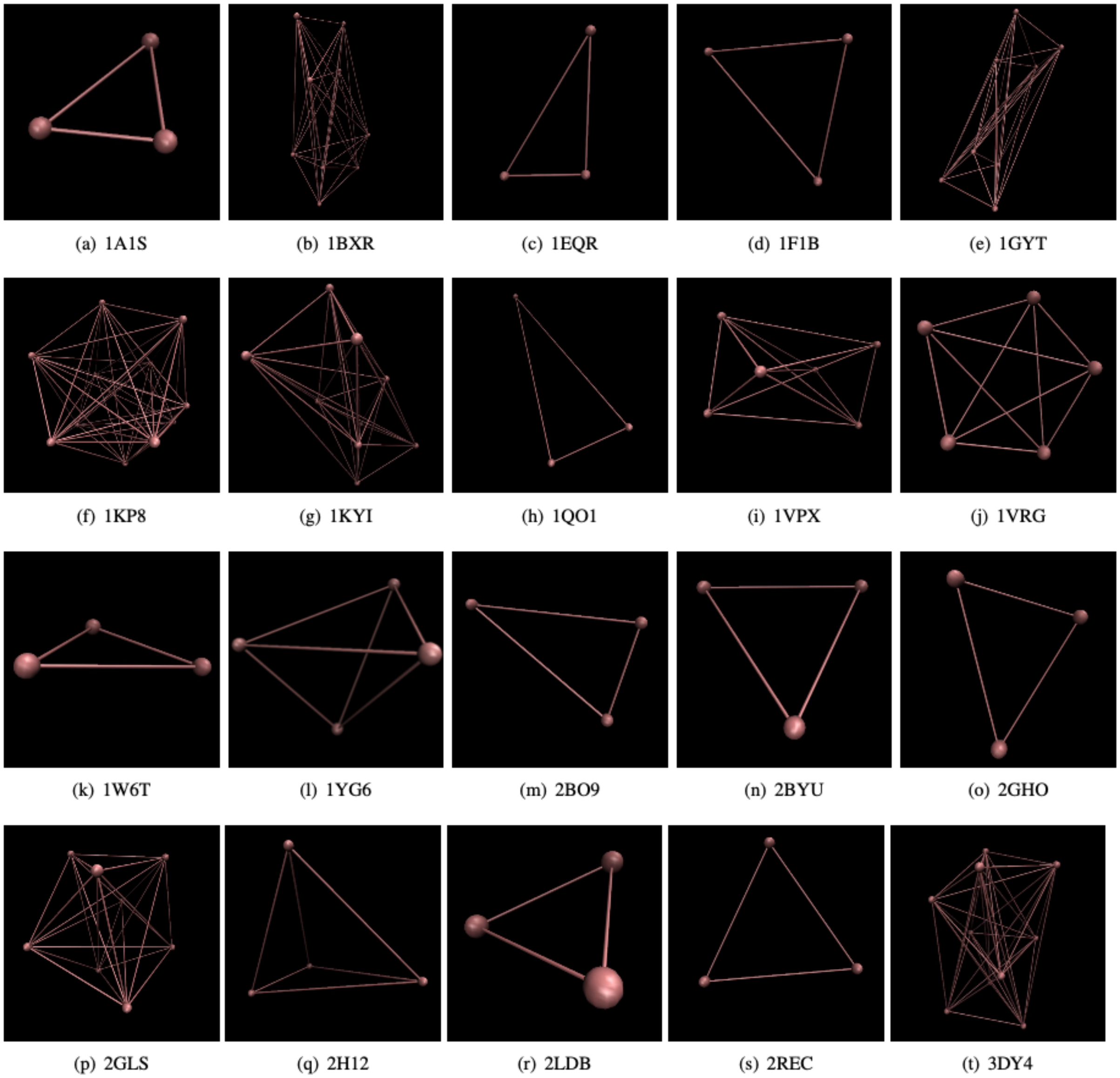

(a) 1A1S (b) 1BXR (c) 1EQR (d) 1F1B (e) 1GYT

(f) 1KP8 (g) 1KYI (h) 1QO1 (i) 1VPX (j) 1VRG

(k) 1W6T (l) 1YG6 (m) 2BO9 (n) 2BYU (o) 2GHO

(p) 2GLS (q) 2H12 (r) 2LDB (s) 2REC (t) 3DY4

Figure S4: The ball stick represent of 20 selected macromolecules' coarse-grained topological structure. All atoms in a macromolecule form a fully connected topology, and each sphere is connected to all the other balls. The bond length of the chemical bond is constant, so the macromolecule will not deform.

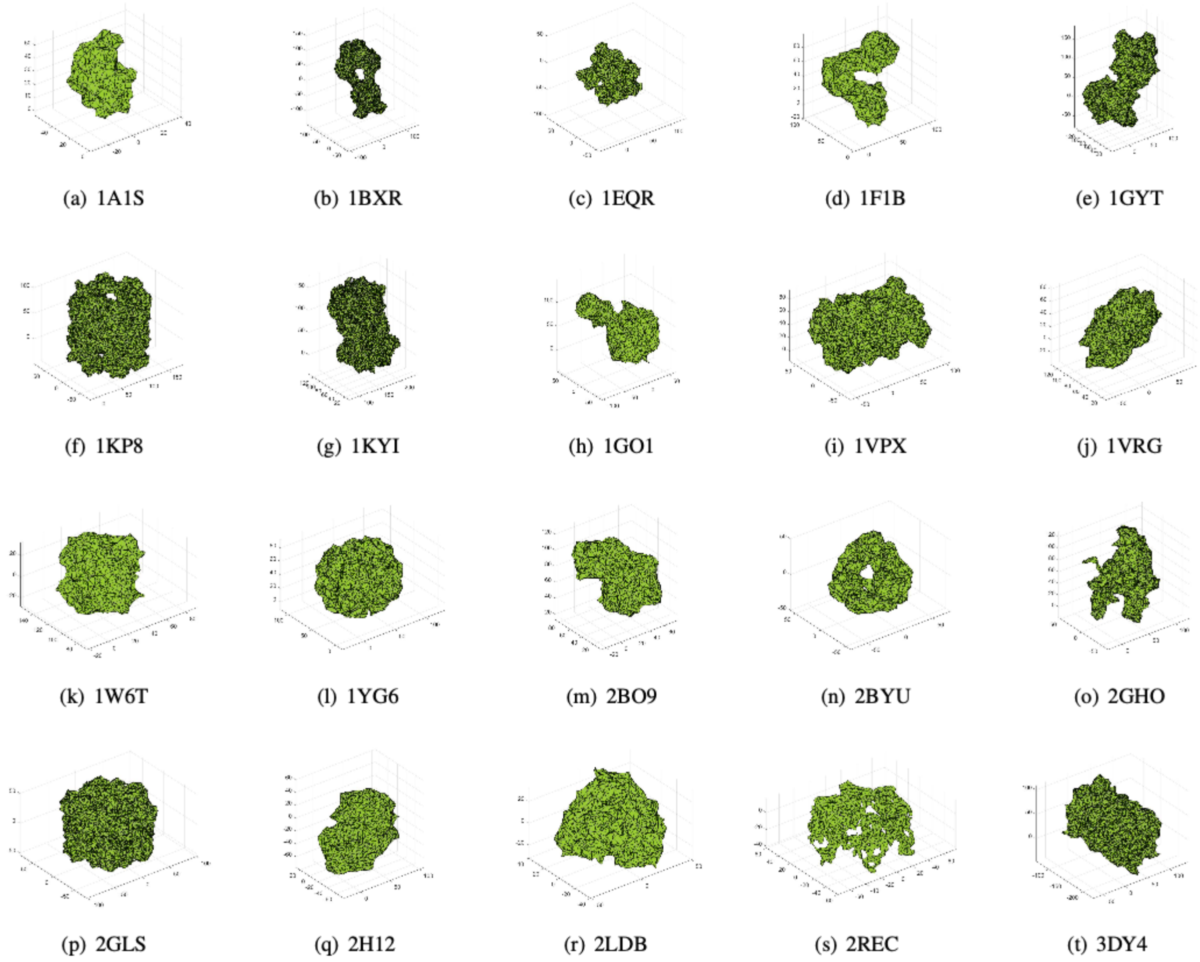

Figure S5: The PDB isosurface of 20 selected macromolecules. Each subfigure is a 3D alpha shape of all the atoms in a macromolecule.

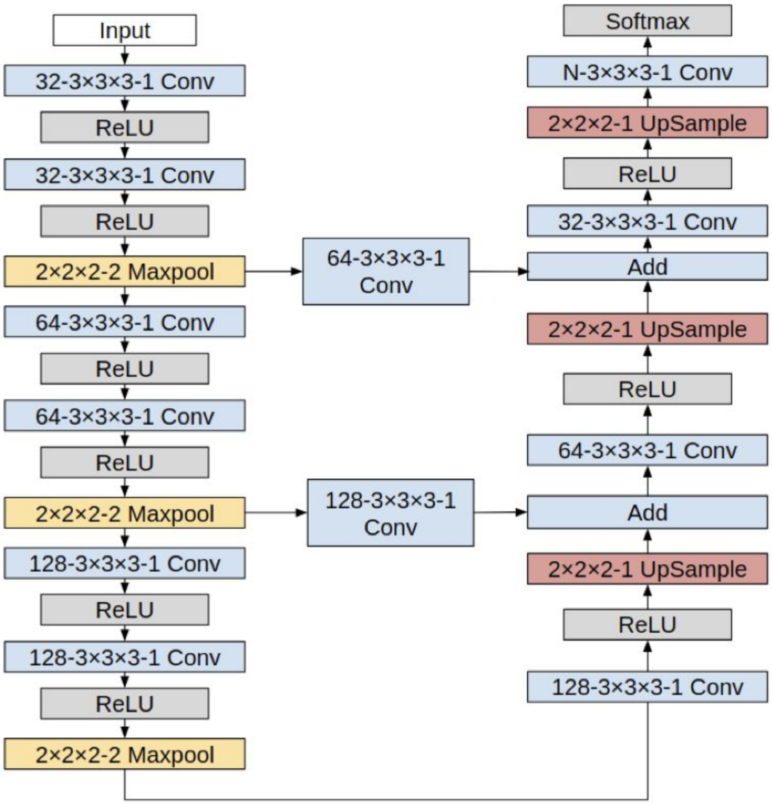

Figure S6: Network architecture of EDSSN3D.
